# Clinical significance of circulating tumor cells and cell‐free DNA in pediatric rhabdomyosarcoma

**DOI:** 10.1002/1878-0261.13197

**Published:** 2022-03-08

**Authors:** Lucia Tombolan, Elisabetta Rossi, Andrea Binatti, Angelica Zin, Mariangela Manicone, Antonella Facchinetti, Silvia Lucchetta, Maria Carmen Affinita, Paolo Bonvini, Stefania Bortoluzzi, Rita Zamarchi, Gianni Bisogno

**Affiliations:** ^1^ Institute of Pediatric Research Fondazione Città della Speranza Padova Italy; ^2^ Department of Woman's and Children's Health, Hematology and Oncology Unit University of Padova Italy; ^3^ Department of Surgery, Oncology and Gastroenterology, Oncology Section University of Padova Italy; ^4^ Veneto Institute of Oncology IOV ‐ IRCCS Padua Italy; ^5^ Department of Molecular Medicine University of Padova Italy

**Keywords:** CellSearch, cfDNA, CTC, RMS, whole‐exome sequencing

## Abstract

Liquid biopsy analysis represents a powerful and noninvasive tool to uncover biomarkers for disseminated disease assessment and longitudinal monitoring of patients. Herein, we explored the value of circulating and disseminated tumor cells (CTC and DTC, respectively) and cell‐free DNA (cfDNA) in pediatric rhabdomyosarcoma (RMS). Peripheral blood and bone marrow samples were analyzed to detect and enumerate CTC and DTC, respectively. We used the epithelial cellular adhesion molecule (EpCAM)‐based CellSearch platform coupled with an automatic device to collect both EpCAM‐positive and EpCAM‐low/negative CTCs. The standard assay was implemented, including the mesenchymal marker desmin. For selected cases, we molecularly profiled primary tumors and liquid biopsy biomarkers using whole‐exome sequencing and droplet digital PCR, respectively. RMS patients with metastatic disease had a significantly higher number of CTCs compared to those with localized disease, whereas DTCs were detected independently of disease presentation. The use of the desmin marker remarkably increased the identification of CTCs and DTCs in RMS samples. Of note, CTC clusters were detected in RMS patients with disseminated disease. Further, cfDNA and CTC molecular features closely reflected the molecular makeup of primary tumors and informed of disease course.

## Graphical abstract

This study shows that circulating tumor cells (CTCs) are detectable in blood and bone marrow of patients with rhabdomyosarcoma and that most CTCs express the mesenchymal marker desmin. We detected CTC clusters and high levels of cell‐free DNA in metastatic rhabdomyosarcoma patients. The concurrent evaluation of cell‐free DNA and CTCs, as well as their molecular characterization, was informative of tumor evolution and therapeutic response.

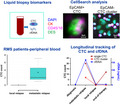

**Abbreviations**
ARMS: alveolar rhabdomyosarcoma
ASCD: automated sample collection device
BM: bone marrow
cfDNA: cell‐free DNA
CK: cytokeratin
CS: CellSearch
CTC: circulating tumor cells
ddPCR: droplet digital PCR
DES: desmin
DTC: disseminated tumor cells
EpCAM: epithelial cell adhesion molecule
ERMS: embryonal rhabdomyosarcoma
PB: peripheral blood
PBMC: peripheral blood mononuclear cells
RMS: rhabdomyosarcoma
VAF: variant allele frequency
WES: whole‐exome sequencing

## 1 Introduction

Rhabdomyosarcoma is the most common soft tissue sarcoma in pediatric patients. It derives from primitive mesenchymal cells that fail to complete myogenesis, though maintaining expression of both desmin and skeletal‐muscle transcription factors myogenin (*MYOG*) and *MYOD1* [1].

Although the survival has improved over the years thanks to a progressive refinement of the treatment, it remains a major cause of death from cancer in children and adolescents. Treatment modalities and intensity are tailored according to a series of prognostic factors, with the presence of metastasis at diagnosis being the most important. In this case, the prognosis is dismal, with < 30% of children surviving 3 years after diagnosis. A similar unsatisfactory outcome is presented by patients who experience a relapse after treatment with an overall survival below 20% [2, 3]. In this context, there is high demand for more powerful methods to assess the potential of cancer cell dissemination and relapse, likewise new methods for efficient monitoring of response to therapy and identification of residual disease. This may improve the management of children at high risk of relapse and ultimately increase patient overall survival.

Liquid biopsy analysis represents a powerful tool to uncover biomarkers useful to address these clinical issues. This minimally invasive approach may provide an alternative and easy access to tumor material like CTCs or tumor cfDNA, at diagnosis and during treatment [4, 5, 6, 7]. It has been proven that dynamic changes of CTC and cfDNA levels in cancer patients reflect tumor dissemination and closely predict the progression of disease [8, 9]. The molecular characterization of CTCs and cell‐free DNA has the additional potential to provide a powerful tool to analyze tumor‐specific alterations in cancer patients and estimate tumor burden and treatment efficacy [6, 10]. In studies of sarcomas in adults, CTCs have been isolated based on their larger size compared with normal blood cells [11] or using beads conjugated with specific tumor markers, such as CD99 in Ewing sarcomas [12]. These studies provided evidence that CTCs can be detected and retrieved from the blood of patients with mesenchymal tumors. Plus, the detection and molecular characterization of cfDNA has been reported in pediatric sarcomas, such as osteosarcoma and Ewing sarcoma, and an inverse correlation between cfDNA levels and outcome has emerged [13]. In neuroblastoma patients, dynamic changes in cfDNA copy number and single‐nucleotide variants, along disease progression, have highlighted the value of cfDNA molecular characterization to unveil tumor heterogeneity and inform of metastatic evolution, perhaps throughout the selection of more aggressive clones [14, 15]. In sarcoma patients, studies focused on molecular characterization of cfDNA only has demonstrated a correlation between changes in levels and response to treatment [16, 17].

In the first study focused on pediatric RMS patients, we extended this notion by demonstrating that CTCs can be detected and enumerated in peripheral blood and bone marrow of children and adolescents with RMS, using the EpCAM‐based CTC enrichment system CellSearch integrated with desmin, a protein widely express by RMS cells [18]. Herein, we obtained quantification of both CTCs and plasma cfDNA, before and during treatment, as well as the CTCs and cfDNA mutational profiles for selected cases. We demonstrated that both CTCs and cfDNA are circulating biomarkers that reflect the molecular makeup of primary RMS tumors and represent promising tools to monitor the disease evolution of affected children.

## 2 Materials and methods

### 2.1 Patients and samples

From December 2015 to February 2019, we collected peripheral blood (PB) and bone marrow (BM) samples from 17 RMS pediatric patients, treated in our Hematology and Oncology Unit, upon signed informed consent. This study conforms to the provisions of the Helsinki Declaration, as revised in 2013, and was approved by the local Ethics Committee (protocol number 3347/AO/14).

Patient clinical features are summarized in Table 1. All patients were treated according to the RMS2005 protocol which includes nine cycles of intensive chemotherapy with or without low‐dose maintenance, surgery delayed, and radiotherapy as local treatment, according to the risk group [19]. Before treatment initiation, primary tumor specimens were analyzed with both immunohistochemistry and molecular biology, for diagnostic purposes. Three patients were included in the study at the moment of relapse and were grouped according to the type of relapse (local or metastatic). Concerning liquid biopsy specimens, the blood draws calendar of the study included serial sampling: at enrollment (baseline, T0) and at the first and subsequent follow‐up visits and/or at clinical progression (T1, T2, and T3). Overall, 29 samples of peripheral blood and 26 samples of bone marrow (site: superior iliac crest, 14 right and 12 left) were examined (Table S1I–II).

**Table 1:** Clinical characteristics of RMS patients.

	Patients
Age, years	
< 1	0
1–9	10
≥ 10	7
Gender	
Female	7
Male	10
Histology	
Alveolar RMS	6
Botroyd RMS	1
Embryonal RMS	10
Fusion status	
Negative	11
Positive	6
Tumor primary site	
Orbit	1
Head and neck (not parameningeal)	1
Parameningeal	3
GU BP	2
GU no BP	2
Extremities	6
Other sites	2
Tumor size	
a: ≤ 5 cm	8
b: > 5 cm	8
ND	1
IRS Group	
IRS I	1
IRS II	3
IRS III	10
IRS IV	3

### 2.2 Cell lines and flow cytometry

Two RMS cell lines (Rh36, RRID:CVCL_M599 and Rh30, RRID: CVCL_0041) for which EpCAM expression was previously proven [18] were assessed for desmin (DES) expression by flow cytometry analysis (Fig. S1A). The recombinant anti‐desmin antibody [clone Y66] (Abcam, Cambridge, UK) was used.

Rh30 cells were obtained from ATCC, whereas Rh36 cells were obtained from Dr. Maria Tsokos (National Cancer Institute, Bethesda) [19]. RMS cells were maintained in Dulbecco’s modified Eagle’s medium comprising 10% fetal bovine serum and penicillin/streptomycin (100 μg·mL^−1^) at 37 °C in 5% CO_2_ in a humidified incubator. RMS cell lines were authenticated using STR profiling, and the experiments were performed with mycoplasma‐free cells.

### 2.3 Development and optimization of CTC assay specific for the expression of the mesenchymal marker desmin in rhabdomyosarcoma cell lines

To explore the ability of antigen‐dependent technology to detect and collect CTCs from blood samples of pediatric RMS patients, the EpCAM‐based CellSearch system equipped with an automated sample collection device (ASCD) was implemented using a specific anti‐desmin monoclonal antibody. To set up the method, two RMS cell lines (Rh36 and Rh30) were assessed for desmin expression by flow cytometry analysis (Fig. S1A) and were spiked into healthy donor whole blood samples at a concentration of 300 cells/7.5 mL and analyzed using the CellSearch/ASCD system to optimize the experimental conditions. The spike‐in concentration was defined according to the literature [20]. RMS cells were obtained from both EpCAM‐positive (CellSearch) and EpCAM‐low/negative (ASCD) fractions (Fig. S1B).

### 2.4 Circulating Tumor Cells and Disseminated Tumor Cells analysis

Peripheral blood and bone marrow samples from each patient were collected into CellSave tubes (Menarini‐Silicon Biosystems, Bologna, Italy). Samples were maintained at room temperature and processed within a maximum of 96 h after collection. The presence of CTCs in PB and DTCs in BM was assessed by the CellSearch System according to the manufacturer’s instructions, with minimal modification [4]. A median volume of 7.5 mL for PB and 3 mL for BM was considered for CTC/DTC enumeration.

CTC or DTC are defined as the events with morphological features of a cell larger than 4 μm in diameter and exhibiting an EpCAM^+^, cytokeratins (CKs) 8^+^, 18^+^, and 19^+^, DAPI^+^, and CD45^‐^ immunophenotype.

The CTC standard assay, developed for epithelial cancer, was implemented with the anti‐desmin antibody (clone Y66) as previously reported for other integrated tests [21, 22]. Therefore, the CellSearch (CS) analysis allows to identify CTCs EpCAM^+^, CK^+^, and DES^+/‐^.

EpCAM‐low/negative cells were collected downstream of the CellSearch processing, using the ASCD. Briefly, ASCD allows collecting the waste of CS procedure, isolating residual discarded tumor cells on a 5‐µm‐pore micro‐sieve membrane (VyCAP, The Netherlands) [20]. The cells were, then, stained for DAPI, CK, and DES and counterstained for CD45/CD16, using a cocktail of fluorescent‐labeled antibodies as previously described for other integrated tests [23]. A total number of CTCs per 7.5 mL of PB or DTCs per 3 mL of BM was calculated, as well as the percentage of DES^+^ cells per sample.

### 2.5 DNA whole genome amplification (WGA) from CTCs

The content of the CellSearch cartridges was harvested and processed for DNA amplification using Ampli1‐WGA Kit (Menarini‐Silicon Biosystems) following the manufacturer’s instructions. Similarly, the ASCD cells were harvested and the DNA was extracted with the QIAmp DNA Micro Kit (QIAGEN, Hilden, Germany). Subsequently, the DNA was amplified using Ampli1‐WGA Kit according to the manufacturer’s instructions. The genomic DNA obtained from CTCs was used as a template in droplet digital PCR (ddPCR) experiments to test specific somatic variants identified by whole‐exome sequencing (WES) on tumor tissue (see next section).

### 2.6 Libraries preparation and whole‐exome sequencing

For whole‐exome sequencing profiling, DNA was extracted from tumor biopsies and peripheral blood mononuclear cells (PBMC) using AllPrep DNA/RNA and protein kit (QIAGEN) following the manufacturer’s instructions. DNA was quantified with Qubit 4 fluorimeter, and the quality was verified by gel electrophoresis. The library preparation was performed by Biodiversa (http://www.biodiversa.it/) using the Agilent SureSelect V6 kit. Paired‐end sequencing (2× 150 bp) was carried out on Illumina HiSeq 4000.

### 2.7 Bioinformatic analysis for variant calling and annotation

Variants from WES were called using iWhale [24], a customizable pipeline that performs bioinformatics analysis of tumor‐control matched samples, from reads alignment to variant calling with Mutect and Mutect2 and annotation. Somatic variants were annotated and prioritized according to population allele frequency (gnomAD < 5%, https://gnomad.broadinstitute.org/) and clinical significance (COSMIC https://cancer.sanger.ac.uk/cosmic, ClinVar https://www.ncbi.nlm.nih.gov/clinvar/, dbSNP https://www.ncbi.nlm.nih.gov/snp/). Five different prediction tools were used to infer the functional impact of variants (SnpEff, FATHMM cancer, CADD, MutPred2 suite, and SilVa). A further prioritization of most likely driver variants was obtained using Cancer Genome Interpreter (http://www.cancergenomeinterpreter.org). The sequencing coverage and quality statistics for each sample are summarized in Table S2.

### 2.8 Isolation of cfDNA

Plasma was obtained from peripheral blood in EDTA tubes. At least 500 µL of plasma were processed to recover cfDNA using QIAamp MinElute ccfDNA Kit (QIAGEN). All steps and optimization phases are described in Tombolan et al. [25]. CfDNA was quantified by the Qubit4 fluorimeter using the High‐Sensitivity DNA assay (HS‐DNA), and the quality assessment was carried out on Agilent Bioanalyzer using dedicated high‐sensitivity chips, to verify the absence of genomic (gDNA) contamination.

### 2.9 Sanger sequencing and Droplet Digital PCR

Specific primers for each genomic region investigated were designed using the primer3 web tool (http://primer3.ut.ee). Then, DNA (50 ng) was amplified using TaqGold reagents (ThermoFisher, USA) in a final volume of 25 µL, following the manufacturer’s instructions. PCR products were enzymatically purified by ExoProStar (Illustra, Mercks), and Sanger sequencing was performed by BMR Genomics (https://www.bmr‐genomics.it).

For each somatic variant analyzed in liquid biopsies, a ddPCR assay was designed by BioRad (Hercules, CA, USA) or IDT (Coralville, IA, USA). The characteristics of primers/probes sets are summarized in Table S3. For each assay, a synthetic DNA (gblock) carrying out the selected somatic variant (positive control) was used to optimize the PCR conditions and to calculate the limit of detection (LOD). The annealing temperature was determined operating a gradient PCR. The BioRad QX200 system was used for ddPCR experiments. In brief, the mix of reaction was prepared using ddPCR Master Mix for Probes (no UNG) and primers/probes at final concentration of 900 nm/250 nm, respectively. As template, 50–100 ng of DNA from tissue and PBMC and 0.3–1 ng of cfDNA were used. The total volume of reaction was fixed to 22 µL. All samples were tested at least in duplicate, and each assay includes also a no‐template control (NTC) and a negative control (DNA from PBMC). Samples are placed into a Manual QX200 Droplet Generator, and then PCR was carried out in a thermal cycler with the following condition: 95 °C for 10 min; 94 °C for 30 s; and 55–60 °C for 1 min (40–50 cycles); 98 °C for 10 min, 4 °C hold. Ramp rate was stetted to 2 °C s^−1^. The PCR plates were analyzed using QX200 Reader and the data analyzed with the quantasoft software (BioRad).

## 3 Results

### 3.1 Patient characteristics

We analyzed a total of 29 PB samples and 26 BM samples from the study cohort of 17 pediatric RMS patients (Table 1 **)**. Samples of 14 patients were collected at diagnosis, while 3 at relapse (2 local and 1 metastatic). The majority of patients evaluated at diagnosis showed a localized disease according to the Intergroup Rhabdomyosarcoma Study grouping system (IRS I‐II‐III, *n* = 11), whereas 3 patients presented metastatic disease (IRS IV). For six patients, serial samples were collected during treatment and follow‐up, as detailed in Table S1I–II. Upon completion of the study, four patients have died for recurrent disease, one remained alive with disease, and 12 were at complete remission with a median follow‐up from diagnosis of 42.1 months (range 32.4–130.8).

### 3.2 Increased CTC detection in pediatric RMS patients using the desmin marker

**Fig. 1:**
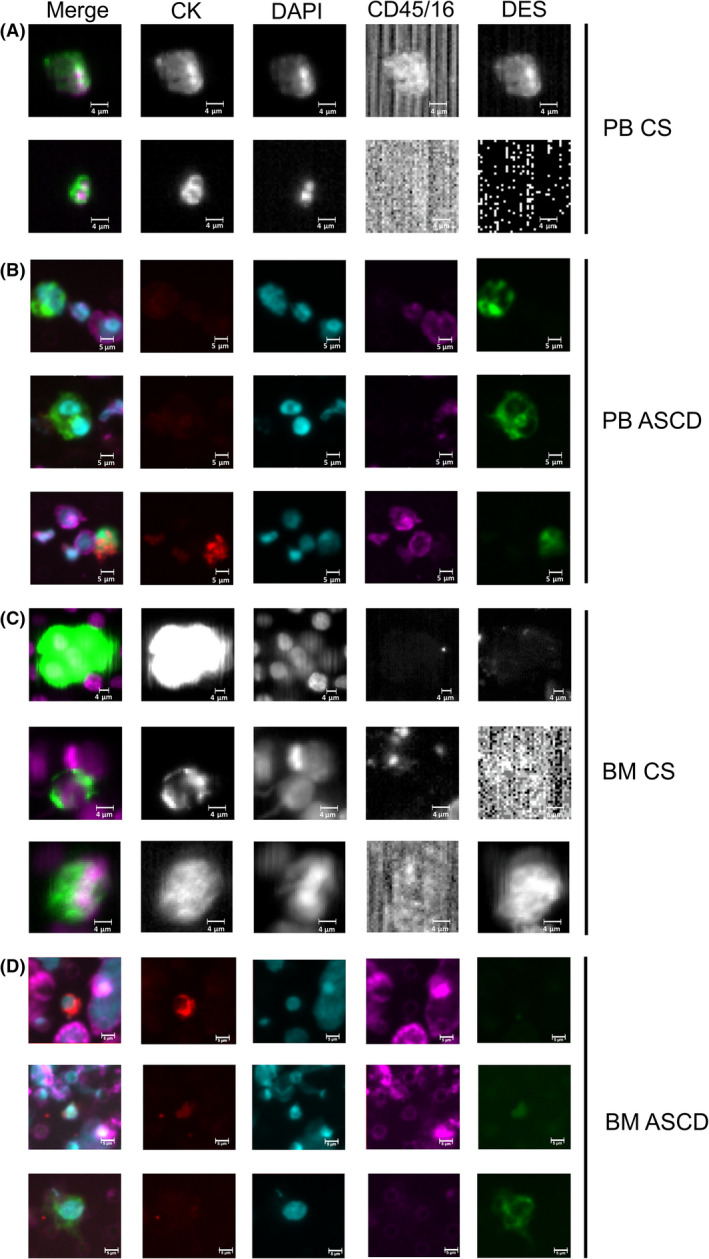
CTC/DTC cells detected in blood and bone marrow of RMS patients. CTCs were identified in peripheral blood (panels A, B) and bone marrow (panels C, D) samples of RMS patients. CTCs were identified in both EpCAM‐positive (CellSearch, CS panels A, C) and EpCAM‐low/negative fraction (microsieves, ASCD panels B, D) and were stained for both cytokeratin (CK) and desmin (DES) markers. Representative CTCs were reported. Cells were stained with DAPI for nuclei and for CD45/16 to avoid leucocytes contamination. First‐column images were obtained by merging all the signals (CK, DES, DAPI, and CD45/16). CTC, circulating tumor cells, DTC, disseminated tumor cells; PB, peripheral blood; BM, bone marrow. Size bar is 4 µm for CS images and 5 µm for ASCD images.

We first optimized a CTC assay specific for the expression of the mesenchymal marker desmin (method section 2.3, Fig. S1), and then we applied the integrated test to PB and BM samples of RMS patients collected prior, during, or after therapy (Fig. 1A,B). Baseline CTC level was assessed in the first blood draw of 13 patients with localized disease and 4 with metastatic RMS (*n* = 3 at diagnosis and 1 at relapse) as reported in Table S4A. When patients with localized disease were considered, 9 out of 13 (69%) had at least 1 CTC, considering both EpCAM‐positive and EpCAM‐low/negative events. Namely, by CellSearch analysis (CS analysis) we found at least one CTC in 7 out of 13 patients (54%) (median 1 CTC, range 1–2), including 4 with at least 1 CTC positive for CK^+^/DES^+^. CK^−^/DES^+^ cells were found only in one sample. Using ASCD, 5 out of 13 patients (38%) had at least one CTC (median value 2 cells, range 1–89): the majority of them (four cases) characterized by CK^−^/DES^+^ cells, and only one presenting also a minority of CK^+^/DES^+^ events (29%). In 3 out of 13 patients with localized disease (23%), both EpCAM‐positive and EpCAM‐low/negative cells were detected, showing higher desmin expression in the latter (median 100%) than in the former fraction (median 0%).

Finally, all RMS patients with metastatic disease at baseline had at least 1 CTC, if we included EpCAM‐positive and/or EpCAM‐low/negative events (Table S4A). Patients with metastatic disease had higher levels of CTCs in the CS fraction compared with patients with localized tumors, though this difference did not reach the statistical significance (Mann–Whitney test *P*‐value = 0.063, Fig. S2A). In 2 out of 3 CTC‐positive cases at least 1 CTC expressed desmin (CK^+^/DES^+^), and in 1 out of 3 of them, CK^‐^/DES^+^ cells were also found.

Using ASCD analysis, all four RMS patients with metastatic disease had at least one CTC (median 14.5, range 1–643), and the majority expressed desmin (median 94.8%, range: 8.3–100%). In line with data obtained by CS analysis, patients with metastatic disease presented a significantly higher number of CTCs compared with cases with localized tumor at ASCD (Mann–Whitney test *P* = 0.0396, Fig. S2B). Importantly, all the cells isolated using the CS/ASCD platform were CD45‐negative and displayed heterogeneous expression of EpCAM, CK, and desmin markers. However, whether such a variable expression correlates with RMS aggressiveness and metastatic potential must be determined.

### 3.3 Detection and enumeration of DTCs in pediatric RMS patients at baseline

We also assessed bone marrow samples in 10 out of 13 (77%) patients with localized disease at baseline (Table S4B,C). In all but one patient (90%), considering both EpCAM‐positive and EpCAM‐low/negative cells, we found at least 1 DTC per 3 mL of BM aspirate (Fig. 1C,D). In particular, CS analysis detected at least 1 DTC at baseline (median 3, range 1–12) in eight cases (80%), with 5 (67%) presenting at least one DES^+^ DTC. ASCD analysis performed in six patients detected at least 1 DTC in 4 of them (median 7, range 4–8), with DES^+^ events found in two.

We also analyzed the BM samples of two RMS patients with metastatic disease at baseline. DTCs were detected in both patients by CS, and most of them were DES^+^ (median 4.5 cell, range 2–7), whereas ASCD analysis detected DTCs in only 1 out of 2 patients with only 5% of cells positive for desmin.

### 3.4 Patients with metastatic disease presented a peculiar CTCs profile

**Fig. 2:**
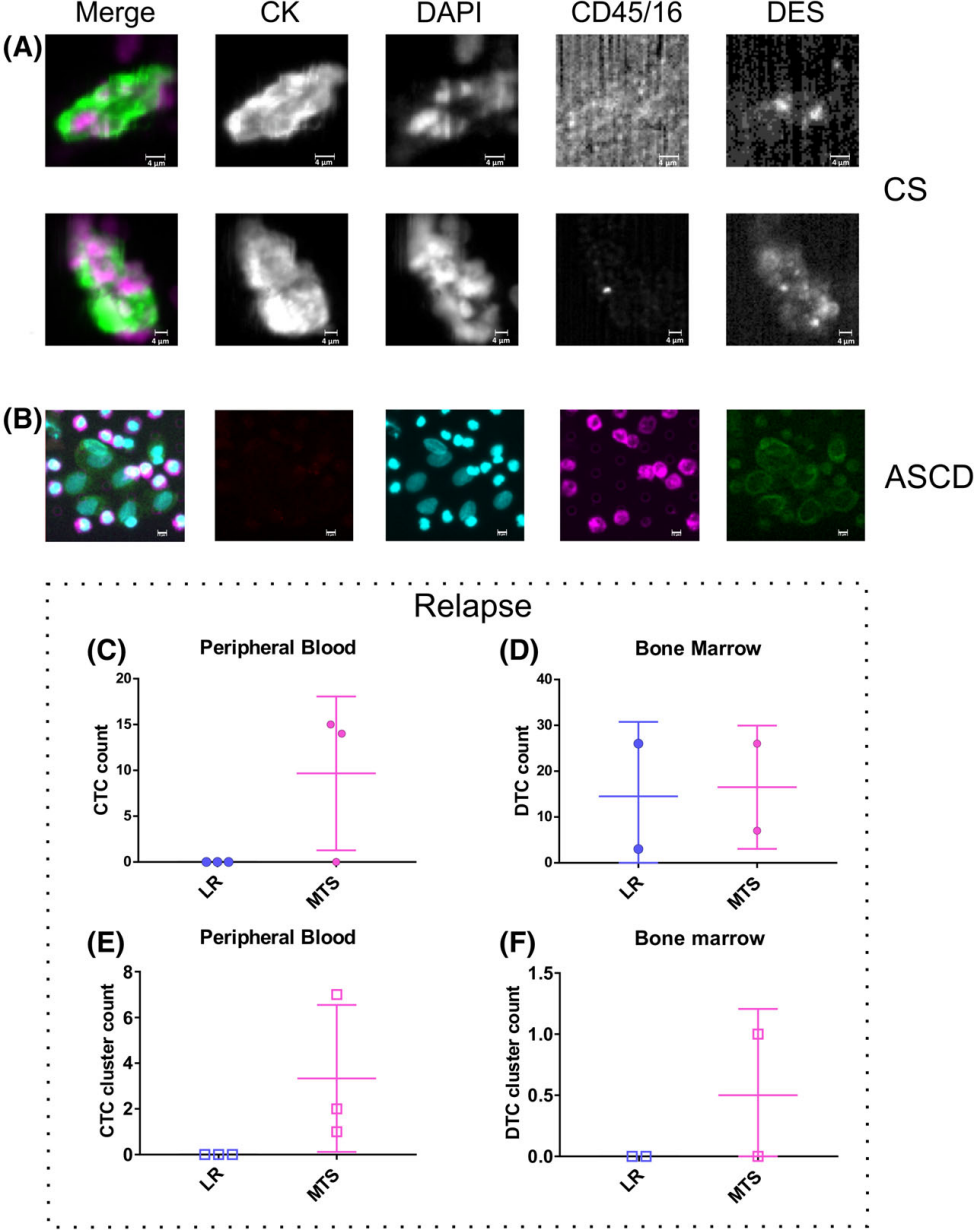
CTC patterns in patients with metastatic RMS disease. CTC clusters were identified in EpCAM‐positive (A) and EpCAM‐low/negative (B) fractions of RMS patients with metastatic disease. Representative CTCs were reported. At recurrence, single cells (C, D) or clusters (E, F), were assessed in blood (C, E) and bone marrow (D, F), comparing cases with local and distal relapses. Nuclei were stained with DAPI dye, while cytoplasm with cytokeratin (CK) and desmin (DES). CD45/16 was used to stain leukocytes. First‐column images in (A, B) are obtained by merging all the signals (CK, DES, DAPI, and CD45/16). CS, CellSearch; ASCD, automated sample collection device. Size bar is 4 µm for CS images and 5 µm for ASCD images.

We next focused on CTC patterns in patients with a disseminated disease, at diagnosis or at relapse. First, we observed that clusters of CTCs, known to be related to an aggressive phenotype in other malignancies [26, 27], were detected in both the patients that had experienced a metastatic relapse (pt#2 and #3). In contrast, two out of three patients with metastatic disease at diagnosis (#4 and #10) showed an elevated number of single CTCs in both EpCAM‐positive and EpCAM‐low/negative fractions, but were negative for CTC clusters. CTC clusters detected using both CS and ASCD systems were all DES^+^ (Fig. 2A,B). In contrast, only one out of 13 patients with localized disease presented a CK^−^/DES^+^ cluster, indicating that CTC clusters are rare when the disease does not spread afar.

An interesting finding also emerged when comparing CTC and DTC profiles in the presence of local or distal relapse. We did not observe either single CTC or CTC clusters in the blood of patients with local relapse (Fig. 2C,E). Plus, single DTCs, but not DTC clusters, were found in the bone marrow of these patients **(**Fig. 2D,F). Therefore, even if the limited number of cases analyzed prevents final conclusions, we speculate that CTC clustering in RMS patients is more frequent in the presence of distal than local relapse.

### 3.5 Somatic mutations in RMS primary tumors detected by WES

Whole‐exome sequencing of eight selected primary tumors and matched PBMC as normal counterpart allowed the identification of the somatic variants present in malignant cells. The molecular characterization of primary tumor samples of patients was useful for the subsequent cfDNA and CTCs analysis. After WES sequence read quality selection and alignment to the reference genome, an average coverage of 138× for tumor and 71× for paired normal samples was obtained, with 85.3% (T = 88.5%, CTR = 82.1%) of the target exome with at least 20× coverage, on average. A total of 747 somatic variants, SNVs and indels, were detected by at least one of the two variant callers used, with an average of 93 variants per patient. After variant annotation and filtering (population allele frequency < 0.05; variant allele frequency—VAF in tumor > 0.05) and analysis with different predictors, 214 high‐confidence somatic variants, in 182 genes, were selected (Table S5). Similar numbers of deleterious variants were observed in cases with embryonal (ERMS, median 18) and alveolar RMS (ARMS, median 21) (Wilcoxon test *P*‐value = 0.79), whereas the second carries pathogenic rearrangements.

Most relevant somatic variants and mutated genes are summarized in Table 2. In the relatively small cohort of patients analyzed, we found somatic variants in genes that, according to the literature, are recurrently altered in the ERMS subtype, including *FGFR4*, *BCOR, KRAS,* and *NF1* [28, 29]. Interestingly, *MAPK* genes (*MAP3K1* and *MAP4K3* *)* variants were found in two fusion‐positive RMS patients and were both predicted as drivers.

**Table 2:** Selected somatic variants identified by WES in primary tumors of eight RMS patients

Patient	Sex	Age	Histology	Variant	Tissue VAF%	Variant type	Variant predicted impact
#1	F	21.9	ARMS+	**MAP3K4 c.4096G** **>A** **p. Gly1366Arg**	20.5	Missense	Protein Kinase domain; Activating
				FES c.2453G>A p. Arg818Gln	39.2	Missense	Protein tyrosine kinase domain; altering protein folding (interaction between K658 and R818)
#2	F	2.4	ERMS	*MCTP1* c.8392A>G	46.5	Splice acceptor variant	Exon skipping variant, loss of C2 domain involved in signal transduction
				*STAG2* c.894‐63T>A	43.3	Intron variant	
#3	M	10.9	ARMS+	***MAP3K1 c.1466C*** **>T** **p. Pro489Leu**	16.9	Missense	Variants hitting zinc finger domain
#4	M	5.1	ERMS	*TEK* c.1004G>A p. Trp335*	19.3	Stop gain	EGF‐like 3 domain; cutting most of the protein
				***FGFR4* c.1648G>C** ***p. Val550Leu***	25.7	Missense	Tyrosine kinase domain; activating variant
				***SMAD3* c.1010‐1G>A**	22.7	Splice acceptor variant	Exon skipping; loss of MH2 domain; altering interactions with other proteins
#6	M	10.3	ERMS	*PGAM2 c.718C>T* *p. Arg240Trp*	50.0	Missense	Gain of acetylation site and loss of methylation site at K241 position; altering interactions with other proteins
				ABCG2 c.1282G>T p. Gly428Trp	47.5	Missense	ABC2 membrane domain; altering interactions with other proteins
				*TMEM104* c.481G>A p. Ala161Thr	46.4	Missense	Transmembrane amino acid transporter domain; loss of an alpha helix
#8	M	4.7	ERMS	***KRAS* c.38G>A** **p. Gly13Asp**	65.1	Missense	Oncogenic variant in various cancer types
				***BCOR* c.724G>T** p. Glu242*	48.2	Stop gain	Loss‐of‐function
				***NF1* c.1549G>T** p. Glu517*	51.5	Stop gain	Both loss‐of‐function (stop before the GTPase activator and CRAL‐TRIO domains)
				***NF1* c.1945G>T** p. Glu649*	55.0	Stop gain	
#9	F	9.2	ERMS	***H3F3A*** **c.344C>G** **p. Ala115Gly**	5.2	Missense	Histone core domain; altering DNA binding
				***TRAF7*** **c.1683C>G** **p. Ser561Arg**	13.1	Missense	WD40 domain; altering protein interactions; altering protein folding
#10	F	11.9	ARMS+	*ARHGAP23 c.2062G>A* *p. Asp688Asn*	8.0	Missense	Loss of proteolytic cleavage at D683 position

Bold text is used for driver mutations.

Moreover, deleterious somatic variants were identified in the tyrosine kinase domains of *TEK* and *FES* proteins. We also found mutations in *SMAD3*, a transcription factor belonging to the TGF‐β signaling pathway that plays a key role in many tumors, and in *MCTP1* gene, whose mutations have been recently associated with acquired drug resistance in carcinomas [30, 31]. Considering the biological function of the mutated genes and the predicted effect of the variants, ten somatic variants with VAF at least 10% in seven primary tumors were selected for validation by Sanger sequencing and were all confirmed (Fig. S3).

### 3.6 Correlation between circulating tumor cells and cell‐free DNA in RMS samples

**Fig. 3:**
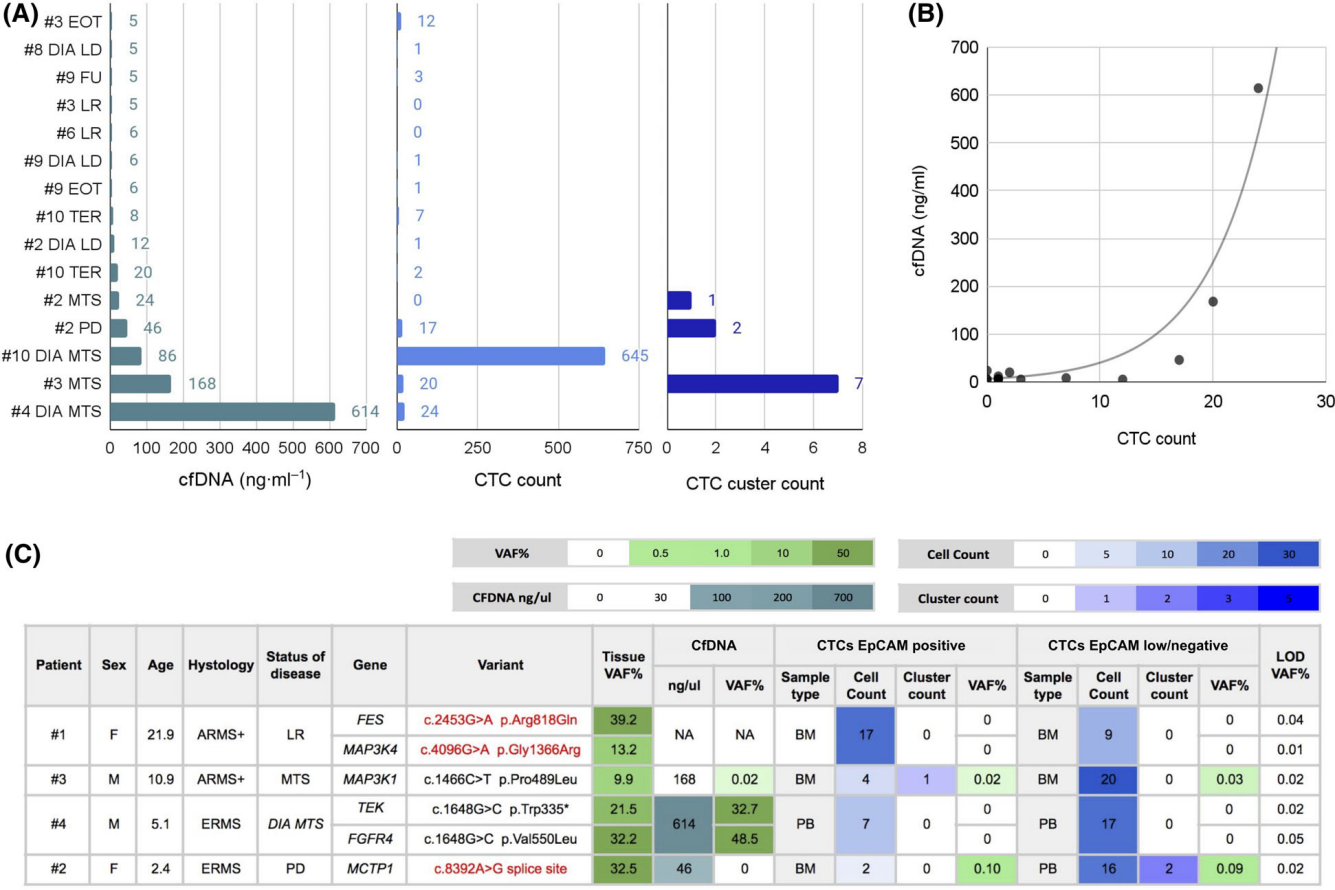
CTCs and cfDNA reflect disease stage and primary tumor molecular profile. (A) CfDNA levels and CTC counts (single cells and clusters) in different RMS patients and blood samples collected at different time points are shown, together with (B) correlation analysis between cfDNA concentration and CTC count. The table below (C) reports the molecular alterations found in primary tumor tissues by WES and validated in liquid biopsies by ddPCR. *DIA LD*, diagnosis of local disease; *LR*, local relapse; *DIA MTS*, metastatic disease at diagnosis; *MTS*, metastasis relapse; *PD*, progressive disease; *TER*, during therapy; *FU*, follow‐up; *EOT*, end of therapy. Sample types used for CTC evaluation: peripheral blood (*PB*) and/or bone marrow (*BM*), NA, sample not available. All samples were tested in ddPCR at least in duplicate. The variant allele frequency (*VAF%*) was calculated by ddPCR software analysis. The limit of detection (*LOD*) VAF% for each assay was calculated using a positive control (gblock).

We analyzed CTCs and cfDNA at different points, corresponding to different disease states, and compared them. Figure 3A shows the amount of cfDNA, CTC, and CTC‐cluster count in peripheral blood of 15 samples. In general, samples with detectable CTCs (single cells or clusters) had also moderate or high levels of cfDNA. Conversely, when no CTCs were detectable, the levels of cfDNA resulted very low and anyhow similar to control samples from healthy donors (HD) (average cfDNA in four HD = 5.37 ng·mL^−1^, range 3.70–10.04, according to literature data [32]). We found a positive correlation (Spearman, *P*‐value 0.09) between CTC count and cfDNA levels in the considered samples, with data well‐fitting (coefficient of determination *R* ^2^ = 0.917) an exponential dependency among CTC count and cfDNA levels (Fig. 3B). Patient #10, due to disseminated disease at diagnosis, multiple sites of metastasis and a number of CTCs much higher than all the other samples analyzed, was not considered for the correlation test.

### 3.7 Molecular characterization of circulating tumor cells and cell‐free DNA

Six somatic variants detected and validated in primary tumors were assessed in liquid biopsies (cfDNA and CTCs amplified DNA) across four RMS patients (Fig. 3C, Fig. S4 **)**, once droplet digital PCR conditions were optimized and the limit of detection (LOD) for each assay determined (see Materials and methods). In this regard, *MAP3K4* and *FES* somatic alterations were detected in the primary tumor but not in CTCs of patient #1 sampled at the time of a local relapse, for which cfDNA was not available. In all the other patients, all the tested variants found in the primary tumor were detected also in liquid biopsy markers. *MAP3K1* variant of patient #3 was detected in both cfDNA and CTC‐DNA, whereas variants of *FGFR4* and *TEK* of patient #4 and *MCTP1* of patient #2 were detected in cfDNA and CTC‐DNA, respectively. Interestingly, the alterations identified in patients #2 and #3 by WES (*MCTP1 and MAP3K1*) were consistently detected in liquid biopsies (peripheral blood or bone marrow) analyzed at different time points of disease evolution.

These findings demonstrate that CTCs and cfDNA isolated from blood and bone marrow of RMS patients reflect the molecular alterations found in primary tumor tissues. Nonetheless, the detection of half of such variants in cfDNA or CTCs suggests that their concurrent evaluation is indicated.

### 3.8 Monitoring tumor progression through circulating tumor cells and cell‐free DNA analysis

**Fig. 4:**
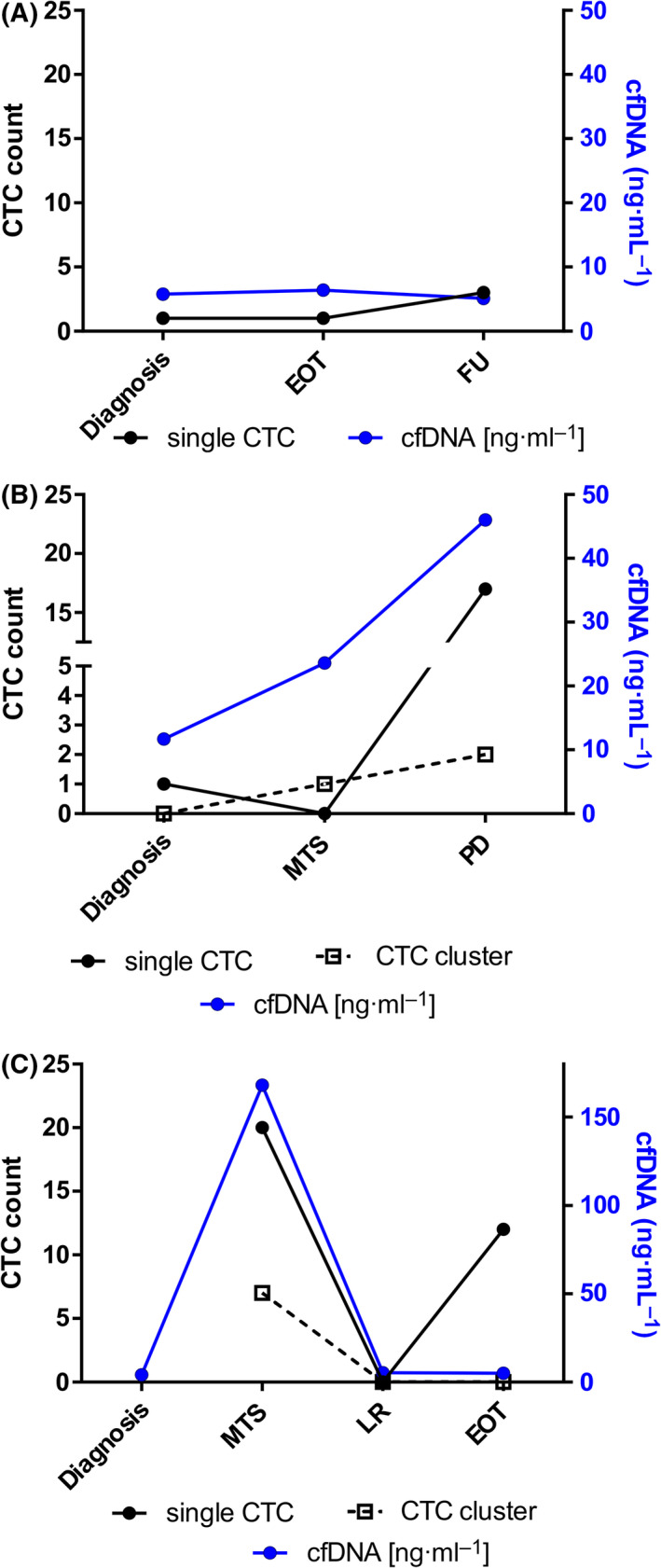
Longitudinal tracking of CTCs and cfDNA in RMS patients. CfDNA and CTCs were analyzed in serial blood samples of three RMS patients, collected at diagnosis and during therapy. (A) Embryonal RMS case with localized disease at diagnosis and IRS I staging (#9), displaying low levels of both CTC and cfDNA over the clinical course of the disease. (B) Localized ERMS tumor (#2) showing low baseline levels of both cfDNA and CTCs, increasing upon distal relapse occurrence and disease progression. (C) Fusion‐positive alveolar RMS patient (#3) displaying dynamic changes of both biomarkers according to the site of relapse occurrence (high with metastatic relapse, *MTS*; low with second local relapse, *LR*). The patient at end of therapy had low levels of cfDNA and a residual CTC count consistent with a clinical condition of complete remission. In black are reported CTCs: single CTCs are represented by circles, whereas CTC clusters by open squares. Each cfDNA quantification was repeated three times. *EOT*, end of therapy; *FU*, follow‐up; *MTS*, metastasis relapse; *LR*, local relapse; *PD* progressive disease.

Longitudinal liquid biopsies analysis has a high potential to monitor the patient’s response to therapy and subclone evolution as well as to uncover disease progression. A series of blood samples collected from the same patient at different time points allowed us to follow liquid biopsy dynamics along disease evolution in a subgroup of six patients (Table S4D–F and Fig. S5). According to the RMS2005 protocol guideline, risk stratification for non‐metastatic RMS depends on clinical prognostic factors such as site of origin, node involvement, size of tumor, and age. Three cases were of particular interest. With this regard, patient #9, with a localized embryonal RMS at diagnosis, was clinically assigned to IRS group I. This case showed at diagnosis low cfDNA level and low CTC count that did not vary during treatment until the end of therapy (EOT). Blood sample collected at follow‐up, 7 months after EOT, confirmed disease remission (Fig. 4A). A different disease course was observed for patient #2, with an embryonal localized tumor at diagnosis but assigned to IRS group III. Accordingly, low levels of both cfDNA and CTC were measured at baseline. At recurrence (14 months from diagnosis; lung involvement), however, an escalation of both parameters was observed and CTC clusters were also detected. The disease progressed further, with a parallel increase in both biomarkers (Fig. 4B). Finally, we tracked cfDNA and CTCs in a fusion‐positive patient (#3) with alveolar RMS and localized disease at diagnosis but classified to IRS group III. Similar to the previous case, cfDNA level was very low at disease presentation while it increased dramatically at first distal relapse (20 months from diagnosis), alongside the number of both single CTCs and clusters. Patient #3 showed a good response to therapy, but relapsed locally within 19 months from the first recurrence. At that time, however, both cfDNA and CTC levels were low or even undetectable. The patient was subjected to surgery and to local radiotherapy, and at the end of treatment, 4 months from the second relapse, the patient showed a low level of cfDNA and a residual CTC count. (Fig. 4C). Since then, patient #3 remained in complete remission (to date, 3 years from EOT), being closely monitored with regular follow‐up controls. Cell‐free DNA level remained stable over the time, with an average of 4.0 ng·mL^−1^ (range between 3.14 and 5 ng·mL^−1^) in three follow‐up samples analyzed.

## 4 Discussion

Despite the good response of primary localized tumors, relapses and progression after chemotherapy such as the occurrence of metastatic disease are very frequent and dramatically worsen the prognosis of children and adolescents affected by RMS. Hence, there is an unmet need for biomarkers able to identify non‐responders and patients at high risk of relapse/progression, perhaps through minimally invasive, easy to perform, and repeatable approaches. Liquid biopsies have the potential of providing the answer to these important clinical needs, as CTCs and cfDNA changes in blood samples of adult cancer patients have been widely accepted and used to correlate tumor response with patient’s outcome [33]. Herein, we investigated the potential and clinical usefulness of CTCs and cfDNA characterization in RMS, an aggressive pediatric mesenchymal tumor, to explore their role as surrogate markers of therapeutic efficacy in anticipating cancer progression. To date, the detection and enumeration of CTCs has been performed extensively in carcinomas [4, 9]. More recently, the chance to detect CTCs from mesenchymal tumors, likewise from epithelial cancers undergoing epithelial‐to‐mesenchymal transition (EMT), has received attention and consideration, as mesenchymal traits have been linked to an increased migration capacity of CTCs and an acquisition of tumor resistance to therapy [34, 35]. In the current study, we analyzed blood and bone marrow samples of pediatric RMS patients, using the CellSearch platform, the only EpCAM‐based CTC enrichment system approved by the FDA for the *in vitro* diagnostic detection and collection of CTCs, coupled to an EpCAM‐independent automatic sample collection device, for the collection of circulating EpCAM‐negative cells as well. We have also implemented the standard CellSearch assay including the mesenchymal marker desmin, which is abundantly expressed in RMS cells but not in blood cells. With this refined methodology, we successfully detected and enumerated CTCs, in the blood and bone marrow of RMS patients, providing the evidence that desmin remarkably increased mainly the EpCAM‐low/negative CTC pool. Few other studies have analyzed the presence of CTCs in blood of sarcoma patients, focusing mainly on adults and considering tumors with different histology, including soft tissue and bone sarcomas. Besides, in these studies cancer patients were not stratified according to the extent of disease [11, 12].

Importantly, this is the first prospective study focused exclusively on pediatric RMS, a rare disease with a limited number of eligible patients and suitable samples, which urgently demands new methods and strategies for anticipating disease dissemination and predicting treatment response. The CTC/cfDNA study of cases with different clinical disease presentations (localized and metastatic disease) is also a novelty for this type of cancer, which demonstrated that a low number of CTCs in blood of children with localized disease or local relapse is likely when compared to that measured in patients with metastatic disease. In contrast, in cases with localized disease that afterward experienced disease progression and relapse, an infiltration of CTCs at diagnosis was always observed, indicating the importance of further exploring CTC quantification as a prognostic marker in these patients. Conversely, bone marrow analysis evidenced DTCs in all cases, supporting the idea that this compartment may represent a site for homing of pediatric RMS cells, and perhaps an early dissemination site. It is known that solid tumors, and in particular rhabdomyosarcoma, can present as micrometastatic diseases, where tumor cells invade peripheral blood and bone marrow [36, 37]. Bone marrow micrometastases are often associated with a significantly higher risk of treatment failure and poorer survival. Therefore, the molecular detection of the minimal disseminated disease is of the utmost importance to identify patients who deserve a better risk stratification and more effective treatment modality [38]. In pediatric RMS, specific molecular markers such as MyoD1 and myogenin and unique fusion transcripts have been used to study disease infiltration in blood and bone marrow, by PCR approaches, since patients with PB positive after treatment and/or BM micrometastases detected by RT‐PCR have a significantly reduced overall survival [37, 39, 40]. Recently, a disseminated disease assessment has been performed in RMS cell lines using QPCR and flow cytometry methods, testing multiple expression markers that have shown the potential to identify CTCs in RMS [41].

Our study aimed at identifying novel biomarkers of liquid biopsy to improve the evaluation of disseminated disease in RMS patients, performing also a molecular characterization of primary tumors and matched circulating tumor cells and cell‐free tumor DNA. Whole‐exome sequencing data on primary tumors confirmed that patients with rhabdomyosarcoma, in particular the alveolar subtype, had a low mutational burden [29]. Anyhow, mutations with a predicted driver role were detected in primary tumors and validated in blood samples. CTC and cfDNA genotyping reflected in part alterations found in matched primary tumors, further suggesting a potential use of them as tracking markers of disease progression over the time. Of importance, our data indicated that the combined molecular characterization of CTCs and cfDNA provides complementary and richer information on tumor biology in individual patients. These findings were supported by the longitudinal analysis of CTC and cfDNA in serially collected blood samples, which demonstrated the value of multi‐parametric blood‐based test to analyze minimal residual RMS disease. Our data are in line with a single‐cell approach in breast cancer, which has shown a variable spectrum of complementary alterations in CTCs and cfDNA obtained from the same patients [42].

Finally, our results revealed a different mode of CTC dissemination in RMS patients who underwent local and distal relapse: the formers characterized by single circulating tumor cells and the latter by multicellular CTC clusters evocative of a collective migration modality found in other metastatic cancers [43].

## 5 Conclusions

We demonstrated the presence of CTCs and DTCs in blood and bone marrow of pediatric RMS patients by using a reliable platform as CellSearch system implemented with desmin marker to detect mesenchymal circulating tumor cells. We observed a different CTC/DTC pattern in local and distal relapsed patients, reporting clusters of CTC only in the presence of metastatic disease. Genomic analysis of primary tumors coupled with validation of selected somatic mutations in matched CTCs and cfDNA confirmed that these two biomarkers have a great potential for disease monitoring in RMS patients as well. Based on this, we recommend the use of multiple biomarkers to improve the prediction of treatment failure and anticipate the cancer progression.

## Conflict of interest

The authors declare no conflict of interest.

## Author contributions

LT contributed to conceptualization, methodology, investigation, and writing—original draft; ER contributed to investigation, methodology, validation, and data Curation; AB contributed to software and formal analysis; AZ and SL involved in investigation; MM and AF involved in investigation and validation; MCA contributed to resources; PB contributed to writing—review and editing; SB involved in formal analysis, visualization, and writing—review and editing; RZ involved in formal analysis, conceptualization, and writing—original draft; GB contributed to supervision, writing—review and editing, and funding acquisition.

### Peer review

The peer review history for this article is available at https://publons.com/publon/10.1002/1878‐0261.13197.

## Supporting information


**Fig. S1**. Optimization of CTC assay with the mesenchymal marker desmin in RMS cell lines.
**Fig. S2**. Comparison of CTC count between localized and metastatic RMS patients.
**Fig. S3**. Validation of WES data by Sanger sequencing.
**Fig. S4**. ddPCR results.
**Fig. S5**. Longitudinal tracking of CTCs and cfDNA in RMS patients—additional info.

**Table S1**. I. Blood draw calendar at baseline. II. Blood draw calendar in serial samples.
**Table S2**. Sequencing coverage and quality of statistics for each sample.
**Table S3**. Summary of the commercial ddPCR primers/probe set used for the experiments.
**Table S4**. CTC count in RMS patients.
**Table S5**. High‐confidence somatic variants detected by WES data analysis.

## Data Availability

The data that supports the findings of this study are available in the supplementary material of this article. Whole‐exome sequencing data are available on request from the corresponding authors. These data are not publicly available due to privacy or ethical restrictions.
