# Supplementary material for: Clinical significance of circulating tumor cells and cell‐free DNA in pediatric rhabdomyosarcoma
Source: Mol Oncol. 2022 Mar 8;16(10):2071–85. doi: 10.1002/1878-0261.13197 (PMC9120897; doi:10.1002/1878-0261.13197)
Supplement: Supplementary file 1 — Fig. S1. Optimization of CTC assay with the mesenchymal marker desmin in RMS cell lines. Fig. S2. Comparison of CTC count between localized and metastatic RMS patients. Fig. S3. Validation of WES data by Sanger sequencing. Fig. S4. ddPCR results. Fig. S5. Longitudinal tracking of CTCs and cfDNA in RMS patients—additional info. [file MOL2-16-2071-s002.pdf]

SUPPLEMENTARY FIGURES

**Supplementary Figure S1.**	Optimization of CTC assay with the mesenchymal marker desmin in RMS cell lines.
**Supplementary Figure S2.**	Comparison of CTC count between localized and metastatic RMS patients.
**Supplementary Figure S3.**	Validation of WES data by Sanger sequencing
**Supplementary Figure S4.**	ddPCR results
**Supplementary Figure S5**.	Longitudinal tracking of CTCs and cfDNA in RMS patients- additional info

FIGURE S1

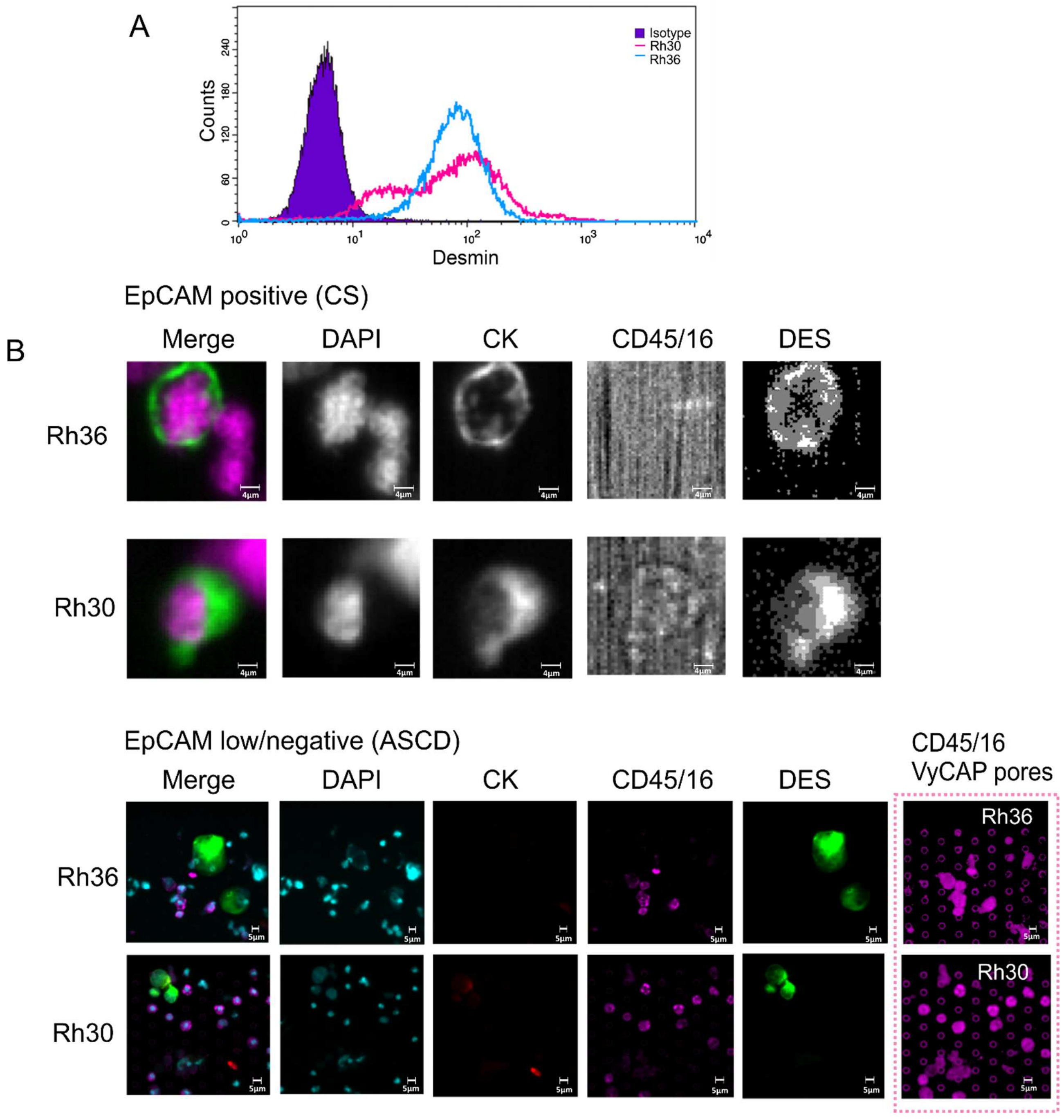

Supplementary Figure S1. Optimization of CTC assay with the mesenchymal marker desmin in RMS cell lines. (**A**) Expression of desmin levels by flow cytometry in Rh36 (blue) and Rh30 (pink) cell lines. In purple the isotype antibody. (**B**) Rh30 and Rh36 cells were spiked into healthy donor whole blood samples and analyzed by both CellSearch (CS) and Automated Sample Collection Device (ASCD). Cells were stained for DAPI, Cytokeratins (CK), leucocyte specific markers (CD45/16) and desmin (DES). In the last column of ASCD panel the images show VyCAP pores. Size bar is 4µm for CS images and 5µm for ASCD images.

FIGURE S2

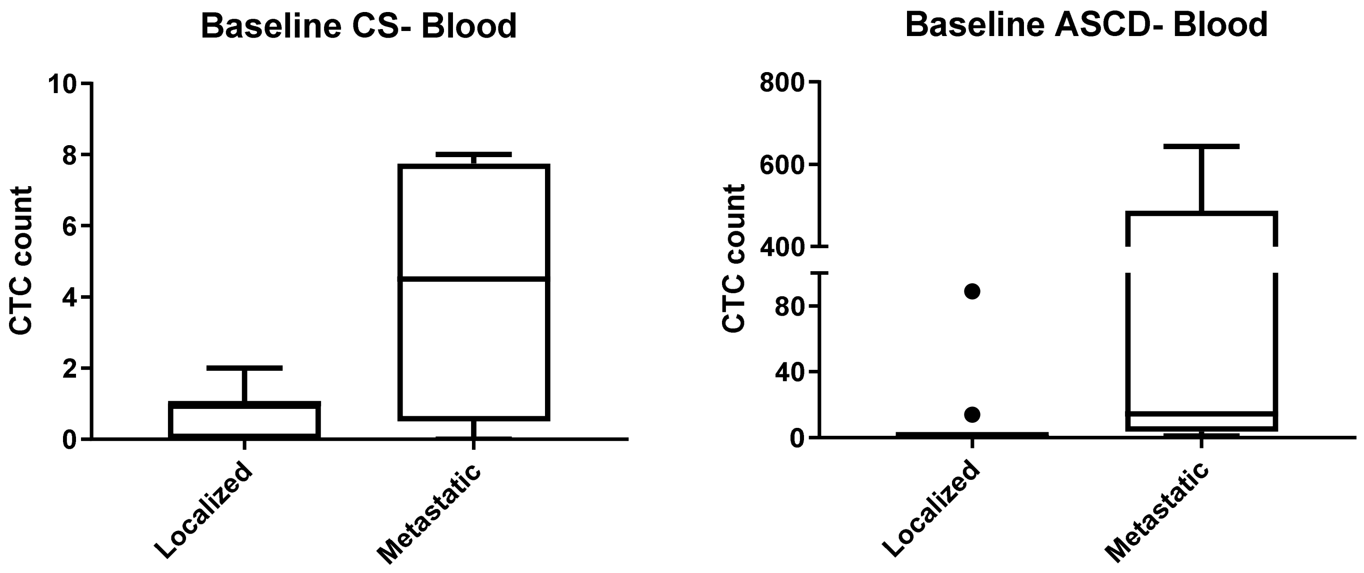

Supplementary Figure S2. Comparison of CTC count between localized(n=13) and metastatic (n=4) RMS patients. (**A**)Box plot relative to CS analysis (Mann-Whitney test p-value= 0.0634); (**B**) Box plot relative to ASCD analysis (Mann-Whitney test p-value= 0.0396). Baseline indicate the first withdrawal for each patient enrolled in the study.

FIGURE S3

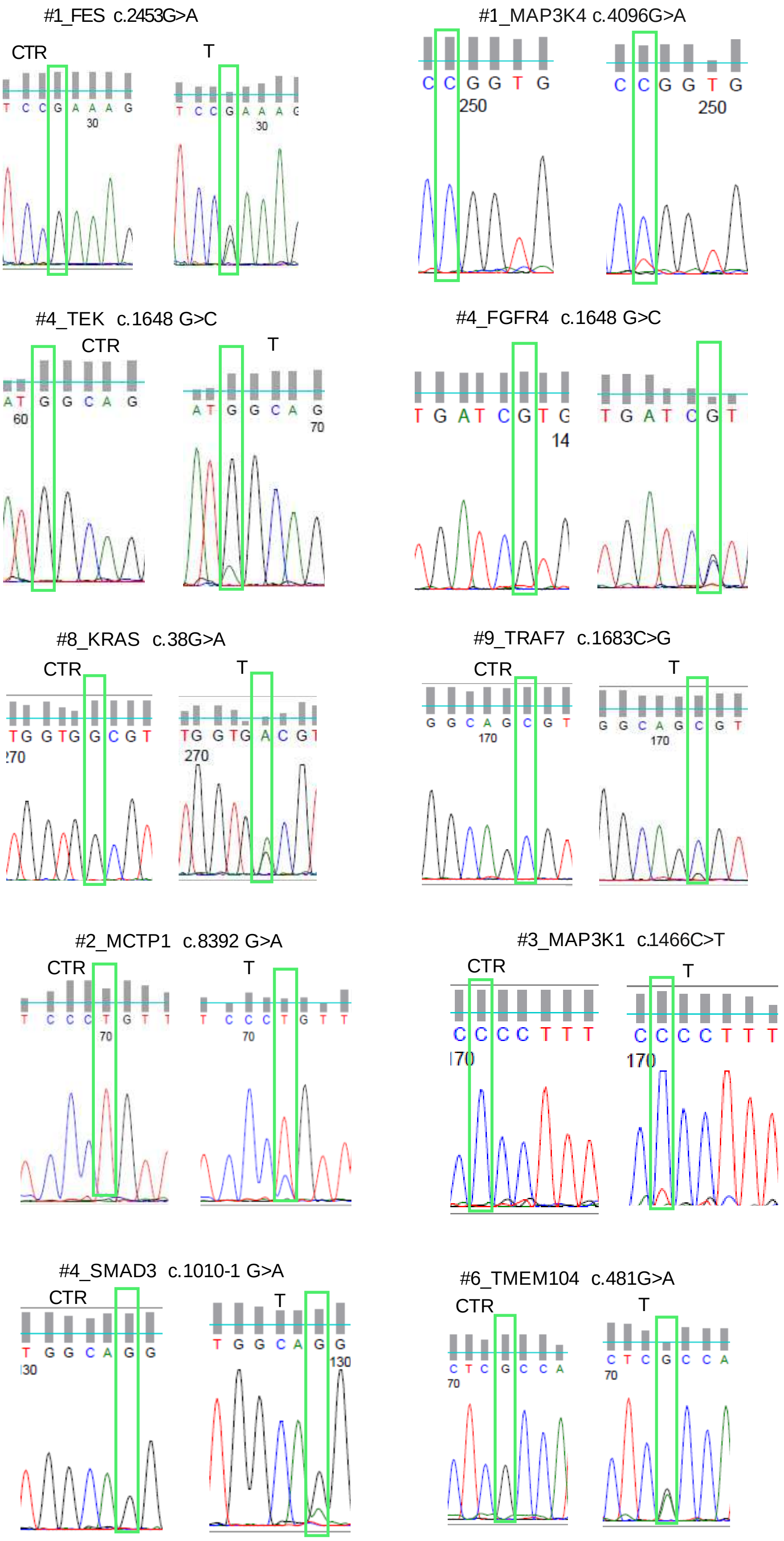

Supplementary Figure S3. Validation of WES data by Sanger sequencing

FIGURE S4

Supplementary Figure S4. ddPCR results (1-D graphs) for the six somatic variants tested in tumor tissue (T) and matched control (CTR) and in liquid biopsies (cfDNA, CTC-DNA). CTC-DNA was amplified using WGA approach described in the methods. CTC/DTC isolated from peripheral blood (PB) and/or bone marrow (BM) were tested. EpCAM+ indicate the cells fraction isolated by CS analysis whereas EpCAM- the cells fraction isolated by ASCD analysis. HEX and FAM are the dyes used in each experiments. wt- wild type; mt- mutated.

FIGURE S4 (cont'd)

A

FES c.2453G>A p.Arg818Gln

1D-graphs

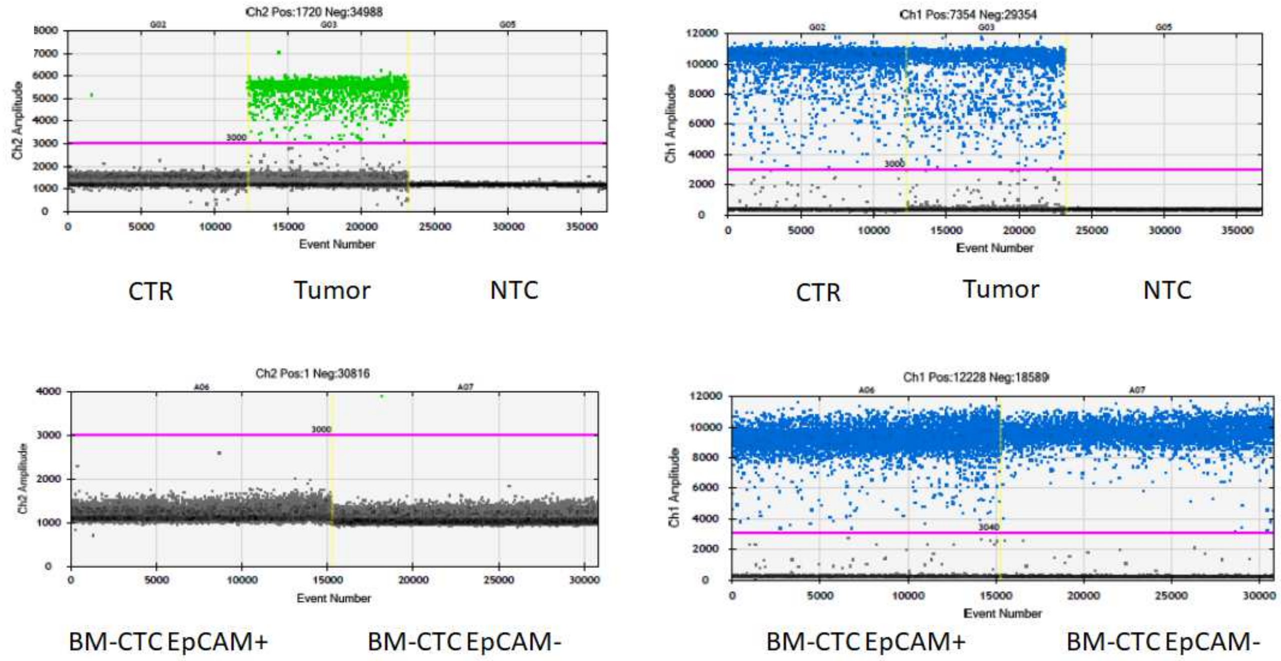

Figure S4

B

MAP3K4 c.4096G>A p.Gly1366Arg

1D-graphs

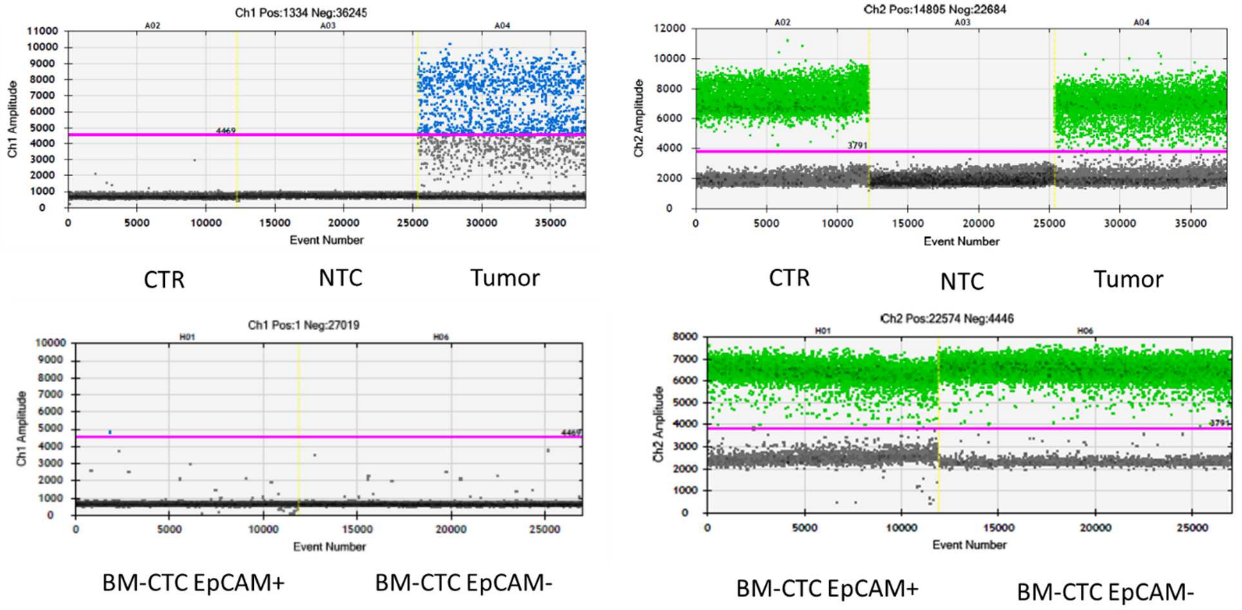

Figure S4

FIGURE S4 (cont'd)

C

MAP3K1 c.1466C>T p.Pro489Leu

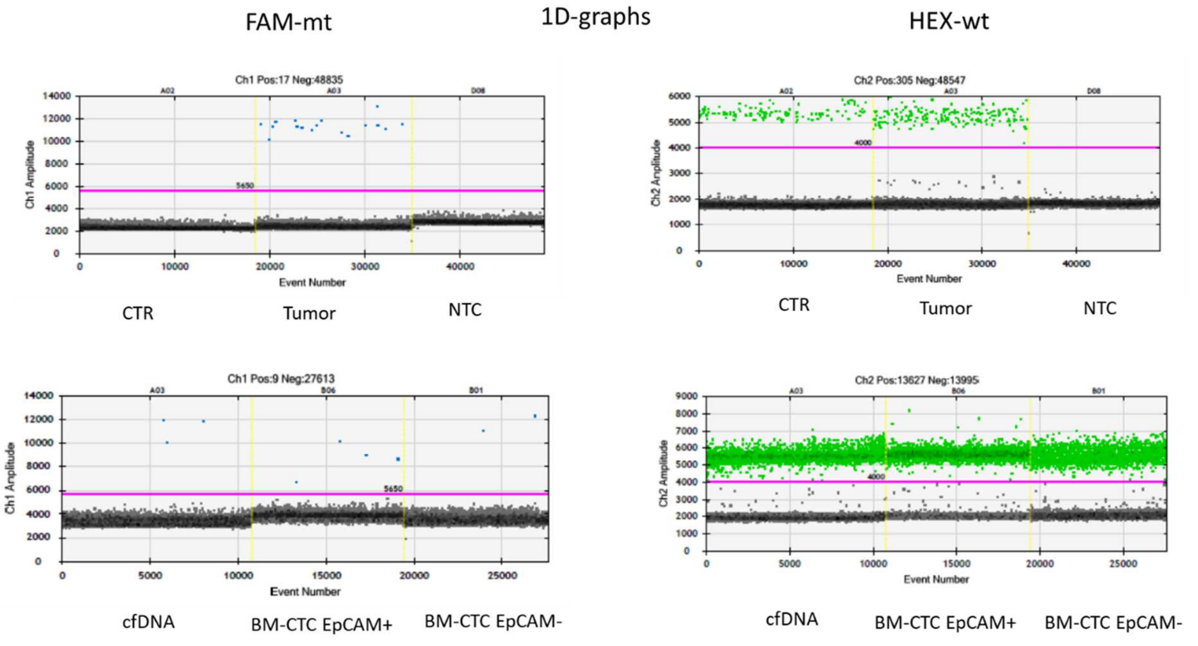

Figure S4

D

TEK c.1004G>A p.Trp335

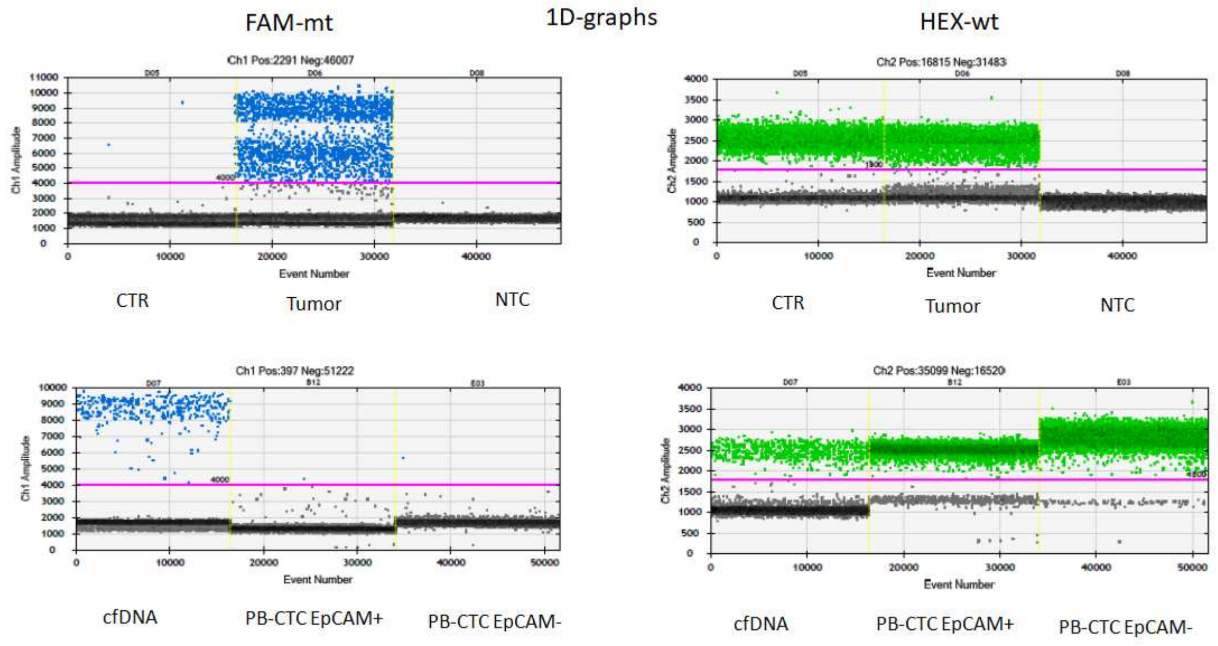

Figure S4

FIGURE S4 (cont'd)

E

FGFR4 c.1648G>C p.Val550Leu

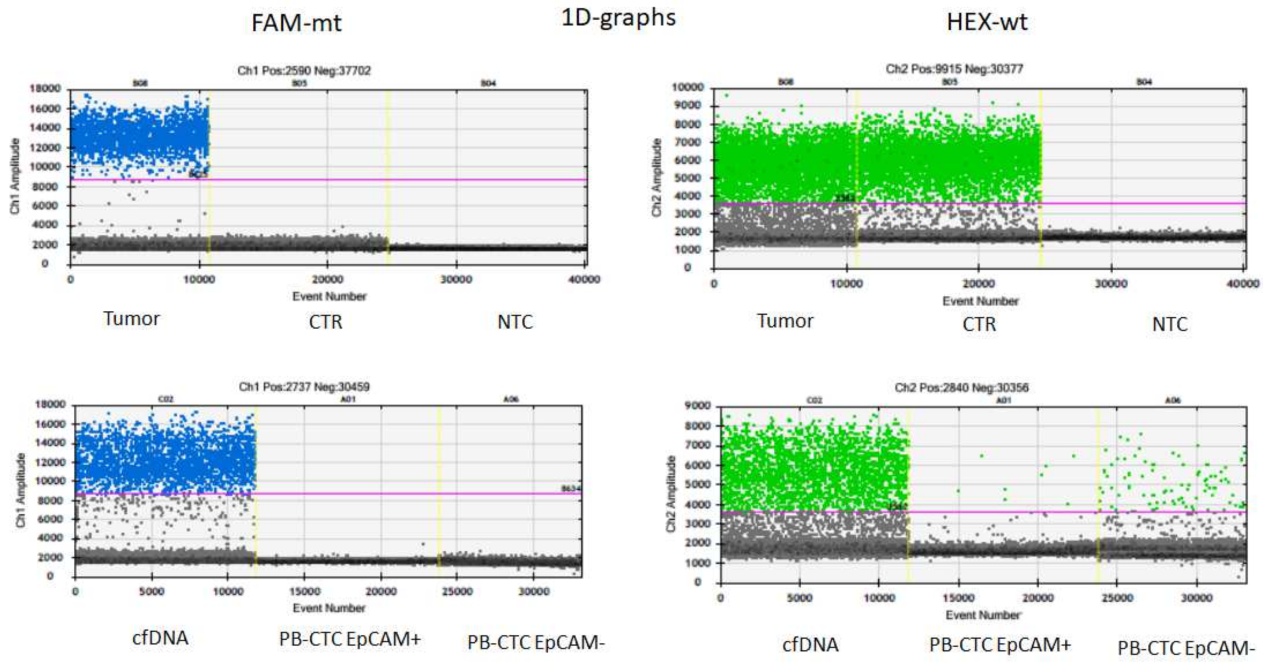

Figure S4

F

MCTP1 c.8392A>G

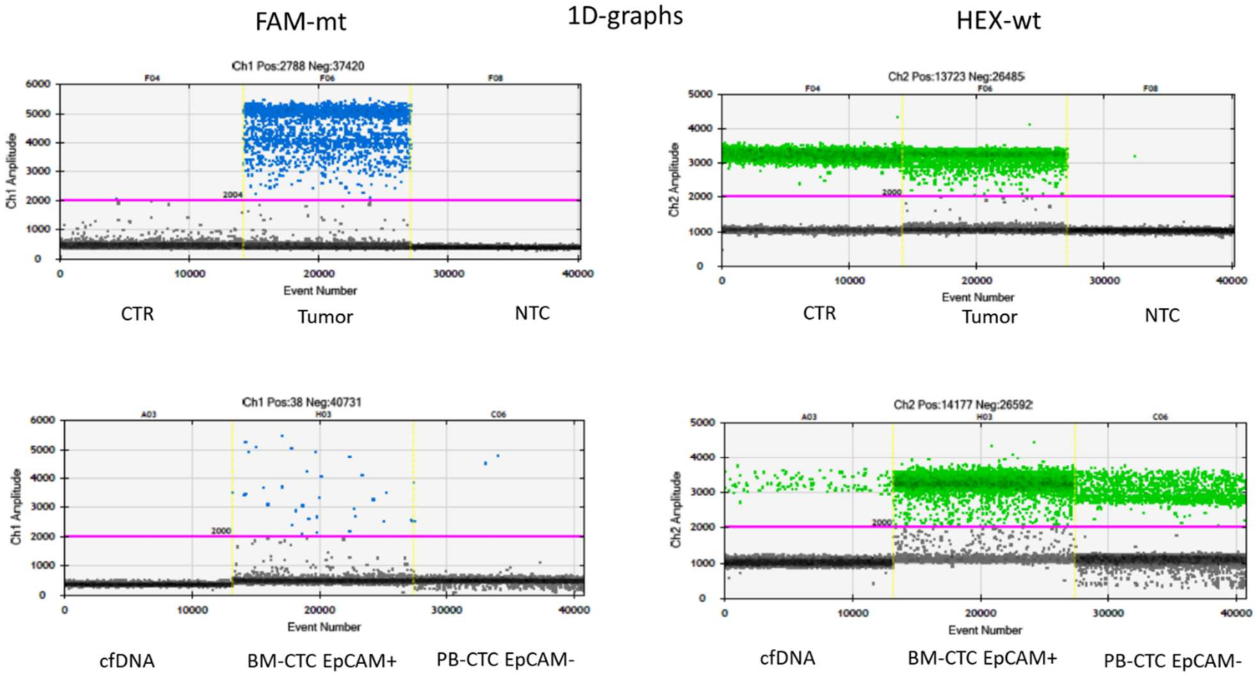

Figure S4

FIGURE S5

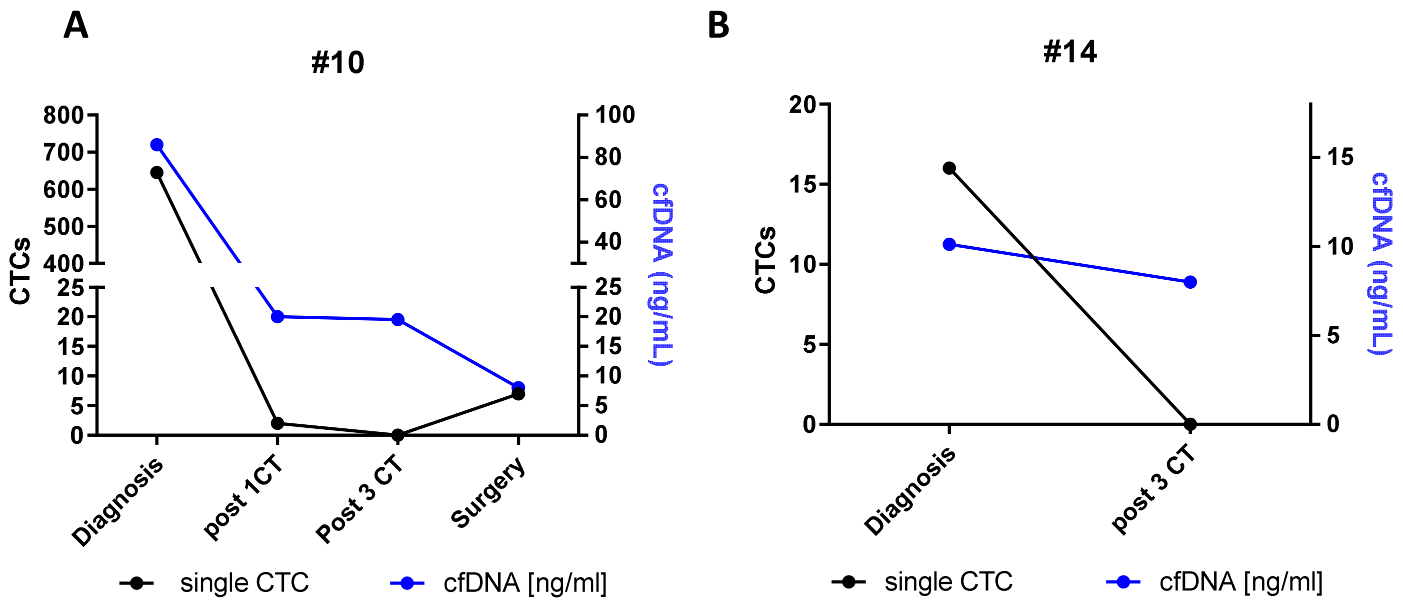

Supplementary Figure S5. Longitudinal tracking of CTCs and cfDNA in RMS patients- additional info. cfDNA and CTCs were analyzed in serial blood samples of other two RMS patients, collected at diagnosis and during therapy. (**A**) Alveolar RMS case with metastatic disease at diagnosis (#10), displaying high levels of both CTC and cfDNA at time of diagnosis. The patient initially response to therapy but unfortunately, we didn't have the opportunity to continue following the patient. (**B**) Localized ERMS tumor (#14) showing basal levels of cfDNA at diagnosis that remain stable during treatment. Conversely, few CTCs was detected at diagnosis but after 3 cycles of chemotherapy the CTCs were undetectable. This patient responded better to chemotherapy and at now is on complete remission. In black are reported CTCs: whereas in blue cfDNA. CT, cycle of chemotherapy.
