# Supplementary material for: Clinical significance of circulating tumor cells and cell‐free DNA in pediatric rhabdomyosarcoma
Source: Mol Oncol. 2022 Mar 8;16(10):2071–85. doi: 10.1002/1878-0261.13197 (PMC9120897; doi:10.1002/1878-0261.13197)
Supplement: Supplementary file 2 — Table S1. I. Blood draw calendar at baseline. II. Blood draw calendar in serial samples. Table S2. Sequencing coverage and quality of statistics for each sample. Table S3. Summary of the commercial ddPCR primers/probe set used for the experiments. Table S4. CTC count in RMS patients. Table S5. High‐confidence somatic variants detected by WES data analysis. [file MOL2-16-2071-s001.pdf]

Supplementary Tables

Supplementary Table S1-I. Blood draw calendar at baseline

Supplementary Table S1-II. Blood draw calendar in serial samples

Supplementary Table S2. Sequencing coverage and quality of statistics for each samples

Supplementary Table S3. Summary of the commercial ddPCR primers/probe set used for the experiments

Supplementary Table S4. CTC count in RMS patients

Supplementary Table S5. High confidence somatic variants detected by WES data analysis

Supplementary Table S1-I. Blood draw calendar at baseline

Patient ID #	age	sex	Date of diagnosis	Histology	IRS	1st blood draw (T0)	Status of disease	CTC	DTC	cfDNA	WES on tumor tissue
1	21	F	27-Sep-10	RMSA	IIa	02-Dec-15	Local Relapse	yes	yes	n.a.	yes
2	2	F	16-Dec-15	RMSE	III	04-Feb-16	Localized disease	yes	yes	yes	yes
3	10	M	24-Sep-14	RMSA	IIIa	11-Apr-16	Metastatic relapse	yes	yes	yes	yes
4	10	M	11-Nov-16	RMSE	IV	17-Nov-16	Metastatic disease	yes	no	yes	yes
5	21	F	1-Nov-16	RMSA	III	18-Nov-16	Localized disease	yes	no	n.a.	no
6	10	M	4-May-16	RMSE	IIa	01-Mar-17	Local Relapse	yes	yes	n.a.	yes
7	30	M	6-Jun-17	RMSA	III	27-Jun-17	Localized disease	yes	yes	n.a.	no
8	3	M	7-Jul-17	RMSE	III	11-Jul-17	Localized disease	yes	no	yes	yes
9	9	F	12-Sep-17	RMSE	I	12-Sep-17	Localized disease	yes	yes	yes	yes
10	11	F	4-Oct-17	RMSA	IV	09-Oct-17	Metastatic disease	yes	no	yes	yes
11	12	F	24-Nov-17	RMSB	II	24-Nov-17	Localized disease	yes	yes	yes	no
12	1	F	27-Dec-17	RMSE	III	18-Jan-18	Localized disease	yes	yes	n.a.	no
13	22	M	14-Feb-18	RMSE	IV	22-Mar-18	Metastatic disease	yes	yes	n.a.	no
14	3	M	06-Apr-18	RMSE	III	30-Mar-18	Localized disease	yes	yes	yes	no
15	25	M	01-Aug-18	RMSA	III	12-Sep-18	Localized disease	yes	yes	n.a.	no
16	10	M	03-Oct-18	RMSE	III	03-Oct-18	Localized disease	yes	yes	yes	no
17	8	M	06-Feb-19	RMSE	III	19-Feb-19	Localized disease	yes	unsuitable	n.a.	no

n.a.= not available

Supplementary Table S1-II. Blood draw calendar in serial samples

Patient ID #	2nd blood draw (T1)	Status of disease	CTC	DTC	cfDNA	3rd blood draw (T2)	Status of disease	CTC	DTC	cfDNA	4th blood draw (T3)	Status of disease	CTC	DTC	cfDNA
1															
2	10-Apr-17	Metastatic relapse	yes	yes	yes	07-Nov-17	Progressive disease	yes	yes	yes					
3	18-Aug-17	Local Relapse suspect	yes		yes	02-Nov-17	Local relapse	yes		yes	21-Mar-18	End of therapy	yes		yes
4															
5															
6															
7															
8															
9	21-Feb-18	EOT	yes		yes	27-Sep-18	FU	yes		yes					
10	30-Oct-17	Post I CT	yes		yes	18-Dec-17	Post III CT	yes		no	19-Jan-18	Surgery	yes		yes
11															
12															
13	14-Sep-18	Progressive disease	yes												
14	09-Jul-18	Post III CT	yes		no										
15															
16															
17															

Supplementary Table S2. Sequencing coverage and quality of statistics for each samples

Sample ID	Total number of sequenced reads	Total number of uniquely mapped non duplicate reads	Total number of covered bases	Median coverage (and range) per base	Percentage of targeted bases with coverage ≥10
#1 tumor	250098081	182048545	119899406	123	97,07%
#1 PBMC	117322367	90415037	119712692	62	94,94%
#2 tumor	61064204	51003209	111546621	31	77,13%
#2 PBMC	65474578	54644896	112613851	35	79,44%
#3 tumor	112251406	83132390	113635861	56	86,08%
#3 PBMC	83533310	67034893	112663347	48	84,17%
#4 tumor	253073977	188615686	120086630	127	97,23%
#4 PBMC	105216583	80207669	119811370	55	93,85%
#6 tumor	206469472	155215271	119992800	109	96,95%
#6 PBMC	99222842	77615700	119737646	55	93,83%
#8 tumor	248241456	182971595	119947344	120	96,81%
#8 PBMC	111631667	87322083	119859282	62	94,85%
#9 tumor	265740876	197018700	119960775	131	97,29%
#9 PBMC	125457721	98267278	119945698	61	95,98%
#10 tumor	198165847	147033477	119726930	102	96,37%
#10 PBMC	92338913	72748573	119663763	51	93,66%

The table summarized the sequencing coverage and quality of statistics for each samples. A Whole-Exome Sequencing data analysis was performed and GRCh37 was used as reference genome.

Supplementary Table S3. Summary of the commercial ddPCR primers/probe set used for the experiments

Gene	Variant	Temperature annealing (°C)	Source	Assay ID	Dye	Amplicon length (bp)
FES	c.2453G>A p.Arg818Gln	60	IDT	306805226 (custom)	Ref FAM;Mut HEX	120
MAP3K4	c.4096G>A p.Gly1366Arg	55	Biorad	dHsaMDS897458835	Ref HEX; Mut FAM	97
MAP3K1	c.1466C>T p.Pro489Leu	55	Biorad	dHsaMDS256514346	Ref HEX; Mut FAM	75
TEK	c.1004G>A p.Trp335*	58	IDT	307569355(custom)	Ref HEX; Mut FAM	93
FGFR4	c.1648G>C p.Val550Leu	55	Biorad	dHsaMDS601206164	Ref HEX; Mut FAM	67
MCTP1	c.8392A>G	60	IDT	314138552 (custom)	Ref HEX; Mut FAM	107

Supplementary Table S4A. CTC count in RMS patients at baseline in peripheral blood (PB)

# patient ID	age	sex	Histology	IRS	Status of disease	Diagnosis	CTC no./7.5 ml PB	% Desm+CTCs	CK-Desmin+ no. /sample	cluster no. of CK+ Desmin+	ASCD no. /sample	% CK+ Desmin+	% CK+ Desmin -	% CK- Desmin+	cluster no. of CK- Desmin+
9	9	F	RMSE	I	Localized	Frontline	1	100			Negative				
11	12	F	RMSB	II	Localized	Frontline	Negative	Negative			Negative				
1	21	F	RMSA	IIa	Localized	Local Relapse	Negative	Negative			Negative				
6	10	M	RMSE	IIa	Localized	Local Relapse	Negative	Negative			Negative				
2	2	F	RMSE	III	Localized	Frontline	1	0			Negative				
5	21	F	RMSA	III	Localized	Frontline	2	100			Negative				
7	30	M	RMSA	III	Localized	Frontline	2	50	1		2			100	1
8	3	M	RMSE	III	Localized	Frontline	Negative	Negative			1			100	
12	1	F	RMSE	III	Localized	Frontline	Negative	Negative			Negative				
14	3	M	RMSE	III	Localized	Frontline	2	0			14			100	
15	25	M	RMSA	III	Localized	Frontline	1	100			Negative				
16	10	M	RMSE	III	Localized	Frontline	1	0			89	29,2		70,8	
17	8	M	RMSE	III	Localized	Frontline	Negative	Negative			2			100	
3	10	M	RMSA	IIIa	Metastatic	Metastatic relapse	8	25		1	12		91,7	8,3	6
4	10	M	RMSE	IV	Metastatic	Frontline	7	28,6	1		17	5,9		94,1	
10	11	F	RMSA	IV	Metastatic	Frontline	2	0			643	4,5		95,5	
13	22	M	RMSE	IV	Metastatic	Frontline	Negative	Negative			1			100	

Supplementary Table S4B. CTC count in RMS patients at baseline in bone marrow_right (BM_right)

# patient ID	age	sex	Histology	IRS	Status of disease	Diagnosis	DTC no. / 4 mL Bone marrow	% Desm+CTCs	CK+Desmin+ no. /sample	cluster CK+ Desm+	cluster CK+ Desm-	ASCD no./ sample	% CK+ Desmin+	% CK+ Desmin -	% CK- Desmin+
9	9	F	RMSE	I	Localized	Frontline	3	33,3							
11	12	F	RMSB	II	Localized	Frontline	Negative	Negative							
1	21	F	RMSA	IIa	Localized	Local Relapse	12	0	0			4		100	
6	10	M	RMSE	IIa	Localized	Local Relapse	3	not done				Negative			
2	2	F	RMSE	III	Localized	Frontline	Negative	Negative				6		100	
5	21	F	RMSA	III	Localized	Frontline									
7	30	M	RMSA	III	Localized	Frontline	1	0							
8	3	M	RMSE	III	Localized	Frontline									
12	1	F	RMSE	III	Localized	Frontline	5	0			1				
14	3	M	RMSE	III	Localized	Frontline	4	25				Negative			
15	25	M	RMSA	III	Localized	Frontline	2	100				8	12,5	87,5	
16	10	M	RMSE	III	Localized	Frontline	1	100				8			100
17	8	M	RMSE	III	Localized	Frontline	Not done	Not done							
3	10	M	RMSA	IIIa	Metastatic	Metastatic relapse	5	20		1		20	5	95	
4	10	M	RMSE	IV	Metastatic	Frontline									
10	11	F	RMSA	IV	Metastatic	Frontline									
13	22	M	RMSE	IV	Metastatic	Frontline	1	100				Negative			

Supplementary Table S4C. CTC count in RMS patients at baseline in bone marrow left (BM_left)

# patient ID	age	sex	Histology	IRS	Status of disease	Diagnosis	DTC no. / 4 mL Bone marrow	% Desm+CTCs	CK-Desmin+ no. /sample	cluster CK+ Desm+	ASCD no. / sample	% CK+ Desmin+	% CK+ Desmin -	% CK- Desmin+
9	9	F	RMSE	I	Localized	Frontline	Negative	Negative						
11	12	F	RMSB	II	Localized	Frontline	Negative	Negative						
1	21	F	RMSA	IIa	Localized	Local Relapse	5	0			5		100	
6	10	M	RMSE	IIa	Localized	Local Relapse	Negative	Negative						
2	2	F	RMSE	III	Localized	Frontline	Negative	Negative			1		100	
5	21	F	RMSA	III	Localized	Frontline								
7	30	M	RMSA	III	Localized	Frontline								
8	3	M	RMSE	III	Localized	Frontline								
12	1	F	RMSE	III	Localized	Frontline								
14	3	M	RMSE	III	Localized	Frontline	3	33,3			Negative			
15	25	M	RMSA	III	Localized	Frontline	Negative	Negative			2		100	
16	10	M	RMSE	III	Localized	Frontline	Negative	Negative			13			100
17	8	M	RMSE	III	Localized	Frontline	Not done	Not done						
3	10	M	RMSA	IIIa	Metastatic	Metastatic relapse	2	100						
4	10	M	RMSE	IV	Metastatic	Frontline								
10	11	F	RMSA	IV	Metastatic	Frontline								
13	22	M	RMSE	IV	Metastatic	Frontline	7	100			Negative			

Supplementary Table S4D. CTC count in RMS patients in serial samples of peripheral blood

# patient ID	age	sex	Histology	2nd Blood draw (T1)	Status of disease	CTC no./7.5 ml PB	% Desm+CTCs	CK-Desmin+ no. /sample	ASCD no. /sample	% CK+ Desmin+	% CK+ Desmin -	% CK- Desmin+	cluster no. of CK- Desmin+
2	2	F	RMSE	10-Apr-17	Metastatic relapse	Negative			Negative				1
3	10	M	RMSA	18-Aug-17	Suspected Local Relapse	1	100		Negative				
9	9	F	RMSE	21-Feb-18	EOT	1	100		Negative				
10	11	F	RMSA	30-Oct-17	Post I CT	2	50		Negative				
13	22	M	RMSE	14-Sep-18	Progressive disease	Negative			negative			-	
14	3	M	RMSE	09-Jul-18	Post III CT	Negative			Negative				

# patient ID	age	sex	Histology	3rd blood draw (T2)	Status of disease	CTC no./7.5 ml PB	% Desm+CTCs	CK-Desmin+ no. /sample	ASCD no. /sample	% CK+ Desmin+	% CK+ Desmin -	% CK- Desmin+	cluster no. of CK- Desmin+
2	2	F	RMSE	07-Nov-17	Metatstatic relapse/Progressive disease	1	100		16	6,3		93,7	2
3	10	M	RMSA	02-Nov-17	Local relapse	Negative			Negative				
9	9	F	RMSE	27-Sep-18	Follow-up	Negative			3			100	
10	11	F	RMSA	18-Dec-17	Post III CT	Negative			Negative				
13	22	M	RMSE										
14	3	M	RMSE										

# patient ID	age	sex	Histology	4th blood draw (T3)	Status of disease	CTC no./7.5 ml PB	% Desm+CTCs	CK-Desmin+ no. /sample	ASCD no. /sample	% CK+ Desmin+	% CK+ Desmin -	% CK- Desmin+	cluster no. of CK- Desmin+
2	2	F	RMSE										
3	10	M	RMSA	21-Mar-18	End of therapy	3	0		9			100	
9	9	F	RMSE										
10	11	F	RMSA	19-Jan-18	Surgery	Negative			7			100	
13	22	M	RMSE										
14	3	M	RMSE										

Supplementary Table S4E-F . CTC count in RMS patients in serial samples of bone marrow right (BM_right) and left (BM-left (BM_left)

# patient ID	age	sex	Histology	2nd BM draw	Status of disease	DTC no./4 ml PB	% Desm+CTCs	CK-Desmin+ no. /sample	ASCD no. /sample	% CK+ Desmin+	% CK+ Desmin -	% CK- Desmin+	cluster no. of CK-Desmin+
2	2	F	RMSE	10-Apr-17	Metastatic relapse	1	0		2	0	50	50	

Supplementary Table S4E_serial samples_BM right

# patient ID	age	sex	Histology	2nd BM draw	Status of disease	DTC no./4 ml PB	% Desm+CTCs	CK-Desmin+ no. /sample	ASCD no. /sample	% CK+ Desmin+	% CK+ Desmin -	% CK- Desmin+	cluster no. of CK-Desmin+
2	2	F	RMSE	10-Apr-17	Metastatic relapse	1	0		3	0	33	67	

Supplementary Table S4F_ serial samples_BM left

Supplementary Table S5. High confidence somatic variants detected by WES data analysis

CHROM	POS	REF	ALT	AF_gnomad	EFFECT	GENE	TRANSCRIPT	CODON_CHANGE	AA_CHANGE	ALLELE_FREQ	SAMPLE
1	17942661	C	T	8,84E-01	missense_variant	ARHGEF10L	ENST00000361221	c.799C>T	p.Arg267Cys	0.182	#4
1	20978298	A	T	.	3_prime_UTR_variant	DDOST	ENST00000375048	c.*574T>A	.	0.349	#10
1	20978298	A	T	.	downstream_gene_variant	PINK1	ENST00000321556	c.*1114A>T	.	0.349	#10
1	56977762	G	T	.	missense_variant	PPAP2B	ENST00000371250	c.696C>A	p.Phe232Leu	0.168	#8
1	85817259	C	A	.	missense_variant	DDAH1	ENST00000284031	c.407G>T	p.Arg136Ile	0.613	#8
1	100340709	G	T	.	splice_acceptor_variant&intron_variant	AGL	ENST00000370163	c.1083-1G>T	.	0.626	#8
1	109952617	G	A	.	missense_variant	PSMA5	ENST00000271308	c.581C>T	p.Ala194Val	0.124	#9
1	116311156	T	A	.	stop_gained	CASQ2	ENST00000261448	c.7A>T	p.Arg3*	0.203	#1
1	145209012	G	A	.	TF_binding_site_variant	.	MA0024.1	n.145209012C>T	.	0.176	#10
1	145209012	G	A	.	TF_binding_site_variant	.	MA0105.1	n.145209012C>T	.	0.176	#10
1	145209012	G	A	.	upstream_gene_variant	NOTCH2NL	ENST00000362074	c.-39845G>A	.	0.176	#10
1	150318967	G	C	.	missense_variant&splice_region_variant	PRPF3	ENST00000324862	c.1843G>C	p.Asp615His	0.166	#1
1	179078473	G	T	.	stop_gained	ABL2	ENST00000502732	c.1929C>A	p.Cys643*	0.319	#8
1	193065785	TAAATAACA	T	.	frameshift_variant	GLRX2	ENST00000367440	c.460_467delTGTTATTT	p.Cys154fs	0.056	#3
1	226259113	C	G	.	missense_variant	H3F3A	ENST00000366813	c.344C>G	p.Ala115Gly	0.052	#9
1	235599868	C	T	2,64E+00	missense_variant	TBCE	ENST00000543662	c.1061C>T	p.Thr354Met	0.105	#6
2	1796223	T	G	.	missense_variant	MYT1L	ENST00000399161	c.3290A>C	p.Glu1097Ala	0.281	#8
2	21246473	C	A	.	missense_variant	APOB	ENST00000233242	c.2528G>T	p.Gly843Val	0.318	#8
2	25656844	C	T	.	synonymous_variant	DTNB	ENST00000406818	c.1278G>A	p.Leu426Leu	0.197	#1
2	46605219	G	A	.	missense_variant	EPAS1	ENST00000263734	c.1436G>A	p.Cys479Tyr	0.269	#8
2	112733032	C	A	.	missense_variant	MERTK	ENST00000421804	c.1127C>A	p.Ala376Asp	0.149	#1
2	140997014	C	A	.	missense_variant	LRP1B	ENST00000389484	c.13412G>T	p.Arg4471Ile	0.184	#4
2	159389694	G	T	.	splice_region_variant	PKP4	ENST00000389759	c.-3G>T	.	0.319	#8
2	159389694	G	T	.	5_prime_UTR_premature_start_codon_gain_variant	PKP4	ENST00000389759	c.-3G>T	.	0.319	#8
2	174094456	G	A	8,84E-01	missense_variant	MLTK	ENST00000409176	c.1027G>A	p.Asp343Asn	0.381	#8
2	220087074	G	GGC	.	frameshift_variant	ATG9A	ENST00000396761	c.1945_1946dupGC	p.Ser650fs	0.215	#1
2	239264638	TCAACAGGTGGA	T	.	splice_acceptor_variant&splice_region_variant&disruptive_inframe_deletion&intron_variant	TRAF3IP1	ENST00000373327	c.1613-5_1618delAACAGGTGGAC	p.Gly538_Leu540delinsVal	0.054	#3
2	239264649	A	ATTTTTTTTTTT	.	frameshift_variant	TRAF3IP1	ENST00000373327	c.1617_1618insTTTTTTTTTTT	p.Leu540fs	0.057	#3
3	33883385	TCAGG	T	.	frameshift_variant&splice_acceptor_variant&splice_region_variant&intron_variant	PDCD6IP	ENST00000307296	c.1182-3_1182delCAGG	p.Val395fs	0.176	#2
3	36873828	G	T	.	intragenic_variant	TRANK1	ENSG00000168016	n.36873828C>A	.	0.488	#8
3	49721606	G	A	3,60E+00	missense_variant	MST1	ENST00000449682	c.2033C>T	p.Pro678Leu	0.083	#2
3	49753432	C	T	8,82E-02	missense_variant	RNF123	ENST00000327697	c.3328C>T	p.Arg1110Cys	0.375	#2
3	51264759	C	A	.	missense_variant	DOCK3	ENST00000266037	c.1423C>A	p.His475Asn	0.262	#4
3	52526232	G	T	.	missense_variant	NISCH	ENST00000479054	c.4249G>T	p.Asp1417Tyr	0.209	#1
3	77526571	G	C	.	missense_variant	ROBO2	ENST00000461745	c.395G>C	p.Arg132Pro	0.49	#8
3	113286549	C	A	.	missense_variant	SIDT1	ENST00000264852	c.507C>A	p.Phe169Leu	0.194	#4
3	129155563	G	GTTTTTT	.	disruptive_inframe_insertion	MBD4	ENST00000249910	c.923_924insAAAAAA	p.Ser308delinsArgLysAsn	0.059	#3
3	172486875	C	A	.	missense_variant	ECT2	ENST00000441497	c.1277C>A	p.Ala426Glu	0.397	#8
3	183955035	G	C	.	missense_variant	VWA5B2	ENST00000426955	c.1555G>C	p.Ala519Pro	0.251	#1
3	186504380	G	T	.	missense_variant	EIF4A2	ENST00000323963	c.717G>T	p.Lys239Asn	0.448	#8
4	738447	G	C	.	missense_variant	PCGF3	ENST00000470161	c.433G>C	p.Asp145His	0.218	#1
4	956351	C	A	.	missense_variant	DGKQ	ENST00000273814	c.2086G>T	p.Asp696Tyr	0.578	#8
4	2883708	G	T	.	missense_variant	ADD1	ENST00000264758	c.279G>T	p.Gln93His	0.432	#8
4	3101084	G	T	.	missense_variant	HTT	ENST00000355072	c.431G>T	p.Arg144Met	0.5	#8
4	26432125	G	T	.	stop_gained	RBPJ	ENST00000342320	c.1126G>T	p.Glu376*	0.541	#8
4	87735600	TGGCCATAG	T	.	frameshift_variant&splice_acceptor_variant&splice_region_variant&intron_variant	PTPN13	ENST00000511467	c.7378-7_7378delGCCATAGG	p.Asp2460fs	0.068	#3
4	87735608	G	GTTTTTTTT	.	frameshift_variant&splice_region_variant	PTPN13	ENST00000511467	c.7378-1_7378insTTTTTTTT	p.Asp2460fs	0.063	#3
4	89022467	C	A	.	missense_variant	ABCG2	ENST00000237612	c.1282G>T	p.Gly428Trp	0.5	#6
4	89602398	C	A	.	missense_variant	HERC3	ENST00000264345	c.2429C>A	p.Pro810Gln	0.498	#8
4	126239854	T	C	.	missense_variant	FAT4	ENST00000394329	c.2288T>C	p.Ile763Thr	0.32	#2
4	190883001	C	G	.	missense_variant	FRG1	ENST00000226798	c.654C>G	p.Asp218Glu	0.056	#8

CHROM	POS	REF	ALT	AF_gnomad	EFFECT	GENE	TRANSCRIPT	CODON_CHANGE	AA_CHANGE	ALLELE_FREQ	SAMPLE
5	33937258	G	T	.	missense_variant	RXFP3	ENST00000330120	c.413G>T	p.Trp138Leu	0.271	#8
5	56168510	C	T	.	missense_variant	MAP3K1	ENST00000399503	c.1466C>T	p.Pro489Leu	0.169	#3
5	61786012	C	A	.	missense_variant	IPO11	ENST00000325324	c.1428C>A	p.Asn476Lys	0.233	#8
5	63991338	G	T	.	missense_variant	FAM159B	ENST00000389074	c.196G>T	p.Gly66Cys	0.303	#8
5	66480130	G	T	.	missense_variant	CD180	ENST00000256447	c.541C>A	p.Gln181Lys	0.305	#8
5	68728357	G	T	.	stop_gained	MARVELD2	ENST00000325631	c.1186G>T	p.Glu396*	0.147	#8
5	94289067	T	C	.	splice_acceptor_variant&intron_variant	MCTP1	ENST00000515393	c.839-2A>G	.	0.465	#2
5	96077047	G	T	.	missense_variant	CAST	ENST00000309190	c.681G>T	p.Lys227Asn	0.249	#8
5	118835081	G	T	.	missense_variant	HSD17B4	ENST00000510025	c.970G>T	p.Ala324Ser	0.111	#2
5	176522544	C	G	9,23E-01	stop_gained	FGFR4	ENST00000502906	c.1641C>G	p.Tyr547*	0.264	#4
5	176522551	G	C	.	missense_variant	FGFR4	ENST00000502906	c.1648G>C	p.Val550Leu	0.257	#4
5	180276208	C	A	.	missense_variant	ZFP62	ENST00000512132	c.2188G>T	p.Gly730Trp	0.233	#8
6	13644858	TC	T	.	frameshift_variant	RANBP9	ENST00000011619	c.1030delG	p.Glu344fs	0.083	#3
6	13644863	C	G	.	missense_variant	RANBP9	ENST00000011619	c.1026G>C	p.Met342Ile	0.079	#3
6	28093480	G	T	.	stop_gained	ZSCAN16	ENST00000340487	c.259G>T	p.Glu87*	0.192	#4
6	41859614	C	A	.	intron_variant	USP49	ENST00000394253	c.-103+2057G>T	.	0.51	#8
6	83898473	C	A	.	missense_variant	PGM3	ENST00000513973	c.249G>T	p.Leu83Phe	0.184	#4
6	90425412	C	A	.	intragenic_variant	MDN1	ENSG00000112159	n.90425412G>T	.	0.422	#8
6	122743299	C	A	.	missense_variant	HSF2	ENST00000368455	c.686C>A	p.Pro229Gln	0.396	#8
6	136667081	G	T	.	missense_variant	MAP7	ENST00000354570	c.2152C>A	p.Pro718Thr	0.419	#8
6	151117014	C	A	.	missense_variant	PLEKHG1	ENST00000358517	c.605C>A	p.Thr202Asn	0.121	#1
6	161528978	G	A	.	missense_variant	MAP3K4	ENST00000392142	c.4096G>A	p.Gly1366Arg	0.205	#1
7	42012198	C	A	.	missense_variant	GLI3	ENST00000395925	c.1841G>T	p.Cys614Phe	0.14	#8
7	44102407	G	A	8,79E-01	missense_variant	PGAM2	ENST00000297283	c.718C>T	p.Arg240Trp	0.475	#6
7	86998748	G	GT	.	frameshift_variant	CROT	ENST00000331536	c.605dupT	p.Phe203fs	0.177	#4
7	107572774	C	T	8,80E-01	missense_variant	LAMB1	ENST00000222399	c.4237G>A	p.Gly1413Arg	0.209	#4
7	121738798	C	G	.	splice_donor_variant&intron_variant	AASS	ENST00000393376	c.1528+1G>C	.	0.327	#2
7	126544155	G	GT	.	frameshift_variant	GRM8	ENST00000339582	c.888dupA	p.Leu297fs	0.132	#10
7	127347672	G	T	.	missense_variant	SND1	ENST00000354725	c.1009G>T	p.Asp337Tyr	0.54	#8
7	151935853	T	C	.	missense_variant	KMT2C	ENST00000355193	c.2591A>G	p.Glu864Gly	0.08	#4
8	17228591	C	A	.	missense_variant	MTMR7	ENST00000180173	c.265G>T	p.Asp89Tyr	0.224	#8
8	28651349	C	A	.	stop_gained	INTS9	ENST00000521022	c.1012G>T	p.Glu338*	0.288	#8
8	52733228	G	A	.	missense_variant	PCMTD1	ENST00000522514	c.757C>T	p.Arg253Cys	0.147	#1
8	52733231	G	A	.	stop_gained	PCMTD1	ENST00000522514	c.754C>T	p.Arg252*	0.052	#1
8	62415934	C	T	.	missense_variant	ASPH	ENST00000379454	c.2261G>A	p.Ser754Asn	0.1	#3
8	67341526	G	T	.	stop_gained	RRS1	ENST00000320270	c.160G>T	p.Gly54*	0.542	#6
8	82370850	TCATCCACAA	T	.	disruptive_inframe_deletion	FABP9	ENST00000379071	c.326_334delTTGTGGATG	p.Ile109_Glu112delinsLys	0.065	#3
8	86118455	G	T	.	missense_variant&splice_region_variant	E2F5	ENST00000416274	c.550G>T	p.Gly184Cys	0.116	#2
8	133645017	C	A	.	intragenic_variant	LRRC6	ENSG00000129295	n.133645017G>T	.	0.241	#8
9	713021	C	T	0.0	missense_variant	KANK1	ENST00000382303	c.2255C>T	p.Ser752Leu	0.236	#4
9	27180340	G	A	.	stop_gained	TEK	ENST00000380036	c.1004G>A	p.Trp335*	0.193	#4
9	95526928	C	CAGCT	.	frameshift_variant	BICD2	ENST00000375512	c.95_98dupAGCT	p.Glu35fs	0.503	#6
9	100136902	G	T	.	stop_gained	CCDC180	ENST00000529487	c.4810G>T	p.Glu1604*	0.533	#8
9	116093304	G	T	.	missense_variant	WDR31	ENST00000341761	c.206C>A	p.Ala69Asp	0.15	#1
9	135271820	C	A	.	intragenic_variant	TTF1	ENSG00000125482	n.135271820G>T	.	0.44	#8
9	140083614	TGA	T	.	frameshift_variant	SSNA1	ENST00000322310	c.150_151delGA	p.Thr51fs	0.487	#6
10	13699351	G	T	.	missense_variant	FRMD4A	ENST00000357447	c.2238C>A	p.Asp746Glu	0.359	#1
10	48413889	A	G	.	intragenic_variant	GDF2	ENSG00000128802	n.48413889T>C	.	0.158	#1
10	60580132	G	A	0.0	missense_variant	BICC1	ENST00000373886	c.2698G>A	p.Asp900Asn	0.435	#1
10	69785304	G	A	.	missense_variant&splice_region_variant	HERC4	ENST00000373700	c.907C>T	p.Arg303Trp	0.449	#8
10	79778994	C	A	.	missense_variant	POLR3A	ENST00000372371	c.1215G>T	p.Arg405Ser	0.587	#8
10	81917520	C	A	.	splice_acceptor_variant&intron_variant	ANXA11	ENST00000372231	c.1336-1G>T	.	0.527	#8
10	96447965	A	T	.	missense_variant	CYP2C18	ENST00000285979	c.415A>T	p.Arg139Trp	0.336	#2
10	122661797	G	T	.	stop_gained	WDR11	ENST00000263461	c.2716G>T	p.Glu906*	0.144	#4

Supplementary Table S5-II

CHROM	POS	REF	ALT	AF_gnomad	EFFECT	GENE	TRANSCRIPT	CODON_CHANGE	AA_CHANGE	ALLELE_FREQ	SAMPLE
11	605118	G	T	.	splice_acceptor_variant&intron_variant	PHRF1	ENST00000416188	c.1153-1G>T	.	0.335	#8
11	4091270	C	A	.	missense_variant	STIM1	ENST00000300737	c.628C>A	p.His210Asn	0.331	#8
11	6486880	C	A	.	missense_variant	TRIM3	ENST00000345851	c.46G>T	p.Asp16Tyr	0.207	#8
11	13408274	G	T	.	missense_variant	ARNTL	ENST00000389707	c.1849G>T	p.Asp617Tyr	0.358	#8
11	13408274	G	T	.	TF_binding_site_variant	.	MA0047.2	n.13408274G>T	.	0.358	#8
11	20409320	G	A	.	missense_variant&splice_region_variant	PRMT3	ENST00000331079	c.28G>A	p.Gly10Ser	0.062	#10
11	34489916	G	C	.	missense_variant	CAT	ENST00000241052	c.1408G>C	p.Ala470Pro	0.189	#4
11	43342407	G	T	.	missense_variant	API5	ENST00000378852	c.268G>T	p.Ala90Ser	0.25	#4
11	47282139	G	A	.	missense_variant	NR1H3	ENST00000467728	c.412G>A	p.Gly138Ser	0.243	#2
11	70007836	G	A	.	missense_variant	ANO1	ENST00000530676	c.1451G>A	p.Gly484Asp	0.26	#1
11	93913149	C	G	.	stop_gained	PANX1	ENST00000227638	c.927C>G	p.Tyr309*	0.265	#4
11	113146029	G	T	.	stop_gained	NCAM1	ENST00000316851	c.2467G>T	p.Glu823*	0.359	#8
11	125889580	G	A	8,81E-01	missense_variant	CDON	ENST00000263577	c.430C>T	p.Pro144Ser	0.061	#8
12	7276744	G	T	.	stop_gained	RBP5	ENST00000266560	c.381C>A	p.Cys127*	0.32	#8
12	7459301	C	A	.	missense_variant	ACSM4	ENST00000399422	c.374C>A	p.Pro125His	0.316	#8
12	25398281	C	T	.	missense_variant	KRAS	ENST00000256078	c.38G>A	p.Gly13Asp	0.651	#8
12	29920851	G	A	.	missense_variant	TMTC1	ENST00000551659	c.460C>T	p.His154Tyr	0.063	#6
12	45173777	G	A	8,81E-01	missense_variant	NELL2	ENST00000429094	c.364C>T	p.Arg122Trp	0.258	#3
12	53470948	G	A	8,79E-01	stop_gained	SPRYD3	ENST00000301463	c.121C>T	p.Arg41*	0.156	#1
12	81028849	C	G	.	intragenic_variant	PTPRQ	ENSG00000139304	n.81028849C>G	.	0.51	#1
12	88589056	G	T	0.0	missense_variant	TMTC3	ENST00000266712	c.2375G>T	p.Arg792Ile	0.348	#8
12	96640998	C	T	8,80E-01	missense_variant	ELK3	ENST00000228741	c.488C>T	p.Thr163Met	0.34	#4
12	98927131	T	C	.	missense_variant	TMPO	ENST00000266732	c.1096T>C	p.Ser366Pro	0.33	#8
12	112681444	C	T	0.0	missense_variant	HECTD4	ENST00000377560	c.4255G>A	p.Gly1419Ser	0.163	#4
12	120784020	G	A	.	missense_variant	MSI1	ENST00000257552	c.965C>T	p.Ala322Val	0.054	#6
12	131622734	C	T	0.0	missense_variant	GPR133	ENST00000261654	c.2489C>T	p.Ser830Phe	0.133	#4
13	20066994	T	C	.	missense_variant	TPTE2	ENST00000390680	c.115A>G	p.Lys39Glu	0.182	#2
13	46354418	C	A	.	3_prime_UTR_variant	SIAH3	ENST00000400405	c.*3100G>T	.	0.196	#8
13	61985776	C	T	1,76E+00	missense_variant	PCDH20	ENST00000409204	c.2456G>A	p.Arg819His	0.295	#8
13	84453618	A	AT	.	frameshift_variant	SLITRK1	ENST00000377084	c.2024dupA	p.Asn675fs	0.456	#6
13	86369440	G	C	.	intragenic_variant	SLITRK6	ENSG00000184564	n.86369440C>G	.	0.82	#3
13	100201431	A	G	.	missense_variant	TM9SF2	ENST00000376387	c.1283A>G	p.Glu428Gly	0.261	#8
13	114438085	G	A	5,16E+00	missense_variant	GRK1	ENST00000335678	c.1441G>A	p.Ala481Thr	0.187	#1
14	24878103	T	C	.	missense_variant	NYNRIN	ENST00000382554	c.1103T>C	p.Phe368Ser	0.194	#3
14	33243084	G	T	.	missense_variant	AKAP6	ENST00000280979	c.3573G>T	p.Lys1191Asn	0.21	#4
14	50666444	G	T	.	missense_variant	SOS2	ENST00000216373	c.475C>A	p.Gln159Lys	0.565	#1
14	60749546	C	A	8,79E-01	missense_variant	PPM1A	ENST00000395076	c.125C>A	p.Ala42Asp	0.333	#8
14	95569894	C	T	.	missense_variant	DICER1	ENST00000527414	c.3839G>A	p.Ser1280Asn	0.221	#1
14	96997920	A	G	.	missense_variant	PAPOLA	ENST00000216277	c.604A>G	p.Asn202Asp	0.357	#8
15	26792973	G	T	.	missense_variant	GABRB3	ENST00000311550	c.1389C>A	p.Phe463Leu	0.517	#8
15	33927854	G	T	.	intragenic_variant	RYR3	ENSG00000198838	n.33927854G>T	.	0.218	#2
15	34395001	G	A	.	missense_variant	PGBD4	ENST00000397766	c.269G>A	p.Gly90Asp	0.069	#4
15	41730541	G	T	.	missense_variant	RTF1	ENST00000389629	c.221G>T	p.Arg74Leu	0.435	#8
15	67479702	G	A	.	splice_acceptor_variant&intron_variant	SMAD3	ENST00000327367	c.1010-1G>A	.	0.227	#4
15	72109951	G	A	.	downstream_gene_variant	MYO9A	ENST00000356056	c.*8970C>T	.	0.241	#4
15	75706628	C	A	.	missense_variant	SIN3A	ENST00000394949	c.391G>T	p.Asp131Tyr	0.107	#8
15	82530669	G	T	.	missense_variant	EFTUD1	ENST00000268206	c.710C>A	p.Ala237Glu	0.483	#8
15	86311412	C	A	.	missense_variant	KLHL25	ENST00000337975	c.1630G>T	p.Gly544Trp	0.492	#8
15	91438772	G	A	.	missense_variant	FES	ENST00000328850	c.2453G>A	p.Arg818Gln	0.392	#1

Supplementary Table S5-III

CHROM	POS	REF	ALT	AF_gnomad	EFFECT	GENE	TRANSCRIPT	CODON_CHANGE	AA_CHANGE	ALLELE_FREQ	SAMPLE
16	202927	G	T	8,88E-01	stop_gained	HBZ	ENST00000252951	c.19G>T	p.Glu7*	0.05	#3
16	202930	A	G	.	missense_variant	HBZ	ENST00000252951	c.22A>G	p.Arg8Gly	0.064	#3
16	555553	G	T	.	missense_variant	RAB11FIP3	ENST00000262305	c.1425G>T	p.Glu475Asp	0.446	#8
16	1756490	G	T	.	missense_variant	MAPK8IP3	ENST00000250894	c.150G>T	p.Glu50Asp	0.427	#8
16	2225891	C	G	.	missense_variant	TRAF7	ENST00000326181	c.1683C>G	p.Ser561Arg	0.131	#9
16	17294506	C	A	.	missense_variant	XYLT1	ENST00000261381	c.919G>T	p.Ala307Ser	0.228	#1
16	21414125	G	T	.	stop_gained	NPIPB3	ENST00000448012	c.2027C>A	p.Ser676*	0.339	#8
16	50368791	G	C	.	missense_variant	BRD7	ENST00000394688	c.718C>G	p.Leu240Val	0.061	#9
16	70211379	C	T	0.0191918	missense_variant	CLEC18C	ENST00000314151	c.452C>T	p.Thr151Met	0.051	#3
17	1782905	G	T	.	missense_variant	RPA1	ENST00000254719	c.1004G>T	p.Arg335Met	0.418	#8
17	3664472	C	A	.	splice_acceptor_variant&intron_variant	ITGAE	ENST00000263087	c.434-1G>T	.	0.522	#8
17	10348314	C	A	.	missense_variant	MYH4	ENST00000255381	c.5445G>T	p.Gln1815His	0.252	#4
17	18906851	C	A	1,11E+00	missense_variant	FAM83G	ENST00000345041	c.504G>T	p.Met168Ile	0.251	#8
17	26905539	C	A	.	missense_variant	SPAG5	ENST00000321765	c.3206G>T	p.Arg1069Ile	0.476	#8
17	29546044	G	T	.	stop_gained	NF1	ENST00000356175	c.1549G>T	p.Glu517*	0.515	#8
17	29552212	G	T	.	stop_gained	NF1	ENST00000356175	c.1945G>T	p.Glu649*	0.55	#8
17	36628191	G	A	.	intragenic_variant	ARHGAP23	ENSG00000225485	n.36628191G>A	.	0.08	#10
17	46048722	C	A	.	splice_region_variant&intron_variant	CDK5RAP3	ENST00000338399	c.7-6C>A	.	0.507	#8
17	46048722	C	A	.	TF_binding_site_variant	.	MA0024.1	n.46048722C>A	.	0.507	#8
17	72791204	G	A	0.0	missense_variant	TMEM104	ENST00000335464	c.481G>A	p.Ala161Thr	0.464	#6
17	74169862	C	T	9,25E-01	missense_variant	RNF157	ENST00000269391	c.217G>A	p.Ala73Thr	0.184	#3
18	23714009	C	T	.	missense_variant	PSMA8	ENST00000308268	c.80C>T	p.Ala27Val	0.063	#4
18	71791808	T	G	.	splice_acceptor_variant&intron_variant	FBXO15	ENST00000419743	c.913-2A>C	.	0.153	#1
19	1054575	C	A	.	missense_variant	ABCA7	ENST00000433129	c.3733C>A	p.Leu1245Met	0.296	#8
19	1223148	A	G	.	missense_variant	STK11	ENST00000326873	c.1085A>G	p.Tyr362Cys	0.337	#1
19	1556584	G	A	.	missense_variant	MEX3D	ENST00000402693	c.934C>T	p.Arg312Cys	0.121	#9
19	11660137	G	T	.	splice_acceptor_variant&intron_variant	CNN1	ENST00000252456	c.502-1G>T	.	0.263	#8
19	12758307	C	A	.	stop_gained	MAN2B1	ENST00000456935	c.2770G>T	p.Gly924*	0.258	#8
19	12903330	G	A	.	missense_variant	JUNB	ENST00000302754	c.745G>A	p.Ala249Thr	0.182	#4
19	14779664	C	T	.	missense_variant&splice_region_variant	EMR3	ENST00000253673	c.26G>A	p.Gly9Asp	0.33	#1
19	15349602	C	A	.	missense_variant	BRD4	ENST00000263377	c.3972G>T	p.Gln1324His	0.159	#2
19	15349602	C	A	.	TF_binding_site_variant	.	MA0139.1	n.15349602G>T	.	0.159	#2
19	15760044	G	T	.	missense_variant	CYP4F3	ENST00000221307	c.600G>T	p.Leu200Phe	0.265	#8
19	23542699	C	T	.	missense_variant	ZNF91	ENST00000300619	c.3082G>A	p.Gly1028Ser	0.254	#8
19	24310121	G	C	.	missense_variant	ZNF254	ENST00000357002	c.1319G>C	p.Gly440Ala	0.267	#4
19	32844002	C	T	.	missense_variant	ZNF507	ENST00000311921	c.266C>T	p.Pro89Leu	0.246	#8
19	42861647	C	A	.	missense_variant	MEGF8	ENST00000334370	c.4721C>A	p.Pro1574Gln	0.204	#8
19	42891511	C	A	.	missense_variant	CNFN	ENST00000597255	c.230G>T	p.Arg77Leu	0.554	#6
19	47219612	A	G	.	missense_variant	PRKD2	ENST00000291281	c.16T>C	p.Ser6Pro	0.14	#4
19	49439374	G	C	0.0	missense_variant	DHDH	ENST00000221403	c.288G>C	p.Glu96Asp	0.197	#4
19	50411870	G	T	.	missense_variant	NUP62	ENST00000422090	c.1195C>A	p.Gln399Lys	0.237	#8
19	51527906	T	G	.	missense_variant	KLK11	ENST00000319720	c.185A>C	p.His62Pro	0.198	#1
19	53014751	T	C	.	missense_variant	ZNF578	ENST00000421239	c.1117T>C	p.Cys373Arg	0.26	#8
19	55795926	C	T	.	missense_variant	BRSK1	ENST00000309383	c.116C>T	p.Thr39Met	0.265	#8
20	8719986	C	G	.	missense_variant	PLCB1	ENST00000338037	c.2287C>G	p.Pro763Ala	0.224	#8
20	31041133	G	A	.	intragenic_variant	C20orf112	ENSG00000197183	n.31041133C>T	.	0.138	#1
20	55940415	G	T	.	missense_variant	RAE1	ENST00000395840	c.292G>T	p.Gly98Trp	0.216	#8
20	61438956	C	T	1,80E-01	missense_variant&splice_region_variant	OGFR	ENST00000290291	c.239C>T	p.Pro80Leu	0.256	#8
20	61987366	G	T	.	missense_variant	CHRNA4	ENST00000370263	c.344C>A	p.Ser115Tyr	0.729	#8
21	45522661	G	T	.	missense_variant&splice_region_variant	TRAPPC10	ENST00000291574	c.3349G>T	p.Val1117Phe	0.269	#8
21	46602599	G	T	.	missense_variant	ADARB1	ENST00000348831	c.1178G>T	p.Cys393Phe	0.254	#8
21	47681298	C	A	.	missense_variant	MCM3AP	ENST00000291688	c.3363G>T	p.Leu1121Phe	0.227	#4
22	19384335	G	A	0.0	missense_variant	HIRA	ENST00000263208	c.629C>T	p.Thr210Ile	0.203	#4
22	20103791	G	T	.	missense_variant	TRMT2A	ENST00000403707	c.369C>A	p.Asp123Glu	0.381	#8
22	21073010	G	C	.	missense_variant	PI4KA	ENST00000255882	c.5217C>G	p.Asn1739Lys	0.257	#1
X	17394343	A	T	.	missense_variant	NHS	ENST00000380060	c.463A>T	p.Ser155Cys	0.259	#2
X	39933875	C	A	.	stop_gained	BCOR	ENST00000397354	c.724G>T	p.Glu242*	0.964	#8
X	47058694	C	T	.	missense_variant	UBA1	ENST00000377351	c.263C>T	p.Ala88Val	0.226	#2
X	77243760	C	A	.	missense_variant	ATP7A	ENST00000341514	c.143C>A	p.Ala48Glu	0.196	#1
X	135582888	A	G	.	missense_variant	HTATSF1	ENST00000535601	c.481A>G	p.Arg161Gly	0.367	#2
X	135591305	A	T	.	missense_variant	HTATSF1	ENST00000535601	c.890A>T	p.Lys297Met	0.385	#2
X	140993505	G	T	.	synonymous_variant	MAGEC1	ENST00000285879	c.315G>T	p.Gly105Gly	0.413	#1

Supplementary Table S5- IV
